# Oncolysis by paramyxoviruses: preclinical and clinical studies

**DOI:** 10.1038/mto.2015.17

**Published:** 2015-10-21

**Authors:** Olga V Matveeva, Zong S Guo, Vyacheslav M Senin, Anna V Senina, Svetlana A Shabalina, Peter M Chumakov

**Affiliations:** 1 Biopolymer Design LLC, Acton, Massachusetts, USA; 2 Engelhardt Institute of Molecular Biology, Moscow, Russia; 3 Division of Surgical Oncology, University of Pittsburgh Cancer Institute, Pittsburgh, Pennsylvania, USA; 4 Blokhin Russian Cancer Research Center, Moscow, Russia; 5 Huntsman Cancer Institute Salt Lake City, Utah, USA; 6 National Center for Biotechnology Information, National Library of Medicine, National Institutes of Health, Bethesda, Maryland, USA

## Abstract

Preclinical studies demonstrate that a broad spectrum of human malignant cells can be killed by oncolytic paramyxoviruses, which include cells of ecto-, endo-, and mesodermal origin. In clinical trials, significant reduction in size or even complete elimination of primary tumors and established metastases are reported. Different routes of viral administration (intratumoral, intravenous, intradermal, intraperitoneal, or intrapleural), and single- versus multiple-dose administration schemes have been explored. The reported side effects are grade 1 and 2, with the most common among them being mild fever. Some advantages in using paramyxoviruses as oncolytic agents versus representatives of other viral families exist. The cytoplasmic replication results in a lack of host genome integration and recombination, which makes paramyxoviruses safer and more attractive candidates for widely used therapeutic oncolysis in comparison with retroviruses or some DNA viruses. The list of oncolytic paramyxovirus representatives includes attenuated measles virus (MV), mumps virus (MuV), low pathogenic Newcastle disease (NDV), and Sendai (SeV) viruses. Metastatic cancer cells frequently overexpress on their surface some molecules that can serve as receptors for MV, MuV, NDV, and SeV. This promotes specific viral attachment to the malignant cell, which is frequently followed by specific viral replication. The paramyxoviruses are capable of inducing efficient syncytium-mediated lyses of cancer cells and elicit strong immunomodulatory effects that dramatically enforce anticancer immune surveillance. In general, preclinical studies and phase 1–3 clinical trials yield very encouraging results and warrant continued research of oncolytic paramyxoviruses as a particularly valuable addition to the existing panel of cancer-fighting approaches.

## Introduction

The idea of using viruses in the treatment of malignancies dates back to the beginning of the 20th century when reports on cases of spontaneous tumor regression after viral diseases or vaccination started to appear.^1–6^ However, it took several decades of intense studies of the complex relations between viruses and their hosts before viruses started to be considered as potential tools for cancer therapy.^7^ Modern studies on oncolytic viruses represent a dynamic and exciting field that absorbs the most recent discoveries in molecular, cell, and cancer biology. Viruses can be quickly modified by recombinant DNA technology thereby rapidly incorporating the fast growing knowledge into oncolytic virus design. The studies involve a wide array of virus species belonging to diverse viral families, such as adenoviruses, herpesviruses, parvoviruses, enteroviruses, reoviruses, rhabdoviruses, paramyxoviruses, myxoviruses, alphaviruses, and poxviruses. Examples of clinical trials include a phase 2 trial of reovirus in combination with chemotherapy in patients with head and neck cancer^8^ and a phase 2 trial of genetically engineered oncolytic poxvirus JX-594 in patients with hepatocellular carcinoma.^9^ These trials confirm that oncolytic viruses do not produce substantial side effects and have considerable antitumor efficacy that affects the overall patient survival.

Recently, the first oncolytic virus treatment in the United States was authorized by the Federal Drug Administration. Government agency approval was granted after completion of phase 1–3 trials that enrolled metastatic melanoma patients. The first oncolytic virus approved by the Federal Drug Administration is a herpes simplex virus-1 construct that encodes human granulocyte-macrophage colony-stimulating factor (GM-CSF).^10^

Innate barriers against viral infection exist at the molecular, cellular, tissue, and organism levels. Ordered tissue organization provides strong protection against virus penetration and spreading. In contrast, cancer cells form disordered structures, which are virus accessible. They lose their duties in the organism and form a separate foreign tissue (tumor) with the sole selfish function of accelerated expansion. The transition makes cells of a tumor a preferable substrate for oncolytic viruses in general and paramyxoviruses in particular (Box 1).

Many viruses have developed mechanisms that modify biosynthetic processes in quiescent cells to stimulate viral replication. Cancer cells already have high metabolic rate and therefore efficiently support propagation of viruses even if they have defects in the host-modifying functions. In response to viral infection, normal cells trigger p53-dependent suicidal programs that kill the abnormal (infected) cells before they start shedding viral progeny.^26–30^ Functional p53 is commonly lost through different mechanisms in cancers^31,32^ and therefore malignant cells frequently do not commit suicide in response to viral infection and efficiently complete virus production.

Virus-infected normal cells start producing interferons (IFNs) that provide antiviral protection for surrounding cells and limit virus spreading. Cancer cells are usually not only defective for the induction of IFNs, but also do not develop resistance to viruses in response to IFN treatment.

In normal cells there is tight crosstalk between the IFN type 1 and p53 pathways. Acting in concert, these two pathways efficiently limit viral infection.^29,33,34^ Loss of p53 functions in cancer cells voids the control mechanisms that ensure their social behavior: the p53-deficient cancer cells can be considered as separate organisms with very unstable genomes that enter the Darwinian competition/selection process. Along with mutations that promote accelerated proliferation and invasion, cancer cells get rid of functions that serve the needs of the whole organism. The induction of IFNs is compromised in multiple tumor types, in particular, through a homozygous loss of chromosome 9p21 region, which encodes type I INF genes.^21^

Frequent loss of type I IFN response in cancer cells is greatly stimulated by the impaired function of the p53 tumor suppressor. As a transcription regulator, p53 is responsible for silencing transcription from LINE and SINE transposons. Inactivation of p53 is associated with a dramatic activation of self-complementary transcripts (double-stranded RNAs) from the widespread repetitive DNA elements such as Alu-repeats.^35^ The double-stranded RNAs elicit a strong type I IFN response that hinders proliferation of cancer cells thereby putting a strong selective pressure and forcing inactivation of the IFN response mechanisms so that further evolution and expansion of cancer cells^35^ is allowed. Cancer cells are generally better hosts for viruses as they acquire defects in the protective mechanisms that resist viral replication. The upregulation of p53 by type I IFNs plays a protective role against the emerging cancer cells.^29,34^ Therefore, cancer cells experience strong selection pressure against both the p53 and the type 1 IFN-mediated mechanisms. During the malignant progression, cancer cells acquire mutations in different components of the IFN system, p53 and apoptotic pathways^32,36–39^ that allow them to escape from the host regulation and to expand. But the very same defects that promote tumor growth provide the opportunity to destroy cancer cells with the use of oncolytic viruses. This also relates to paramyxoviruses.

For example, the oncolytic potential of measles vaccine virus (MV) in a few sarcoma cell lines was negatively correlated with upregulation of expression of RIG-I molecule and the IFN-stimulated gene *IFIT1*. In the MV resistant cell lines, inhibition of viral replication was going along with strong expression of these molecules. In contrast, susceptible cell lines showed a much weaker expression of IFIT1. Pretreatment with IFN-β made the susceptible cell lines more resistant to MV-mediated oncolysis.^40^

However, the preferable killing of cancer cells directly by a virus is not the only mechanism of viral oncolysis. Viruses are potent inducers of innate and adaptive immune responses that greatly contribute to the process.^41–43^

Both innate and adaptive branches of immune system act against viral spreading and persistence in the organism, which in case of oncolytic virus therapy might impose a problem. However, host immune system also recognizes and attacks cancerous cells, and viruses can greatly stimulate the process. Preclinical and clinical data suggest that oncolytic viruses can be regarded as an efficient approach to cancer immunotherapy.^44,45^ Introduction of a nonpathogenic virus strain stimulates natural immune surveillance mechanisms triggering the immune system to recognize and attack malignant cells.^46^

Many viruses, bacteria, and other pathogens have a tendency to accumulate in primary tumors and metastases.^47^ While accumulated in tumors, pathogens bring into any tumor mass a lot of biological materials which is rich with foreign antigens, pathogenic RNA or DNA, and promote multiple danger signals with immuno-triggering properties. In other words, appearance of foreign proteins and genetic material in the malignant cells or near these cells in tumor masses could increase these cells visibility for the immune system.^47^ Numerous cytokines, including IFNs, induced by the pathogen and virus in particular, stimulate antigen processing in immunoproteasomes and presentation of cancer specific epitopes by major histocompatability complex molecules.^48^

Some oncolytic paramyxoviruses might have particular high affinity to cancer cells due to overexpression of viral receptors on their surface. NDV, mumps, and SeV use sialic acid–containing sialoglycoproteins as the cell surface receptors.^49,50^ The abundant presence of sialoglycoproteins on the surface of cancer cells^13,51^ most likely promotes preferential association of the virus with malignant rather than with normal cells and contributes to their selective cytolytic effect in primary tumors and in metastases. UV-inactivated SeV is able to kill prostate carcinoma cells but unable to kill normal prostate epithelium cells. Perhaps, high sensitivity to SeV-mediated cell death of sialic acid–rich prostate carcinoma cells in comparison with normal prostate epithelium is explained by SeV’s preferential association with malignant cells.^52^

The attenuated measles virus (MV Edmonston strain) uses the CD46 receptor, which is a regulator of complement activation that is universally expressed in all nucleated human cells but is often overexpressed in tumor cells.^53^ MV Edmonston strain can kill cells that overexpress this receptor without significant cytopathic effect against nontransformed cells expressing low receptor levels.^54^ Nectin 4 has been identified as the additional receptor for MV.^19^ It is a member of adhesion receptors of the immunoglobulin super-family localized to the adherents’ junctions of epithelial cells. Nectin 4 can be considered as a tumor cell marker for breast,^55^ lung,^16^ and ovarian cancers,^17^ suggesting that it can be partially responsible for the selectivity of MV toward cancer cells.

Are there any advantages in using paramyxoviruses versus representatives of other viral families as oncolytic agents? Recently published review^56^ analyzes these advantages in detail, while a brief description of some of the advantages is provided below.

A potential unique advantage of some paramyxoviruses is the sialidase (neuraminidase) activity of their HN protein, which has the potential to remove sialic residues from the surface of tumor cells.^57,58^ Metastatic cancer cells often express a high density of sialic acid-rich glycoproteins that increase the invasive potential.^13^

One of the possible mechanisms linking the increased sialylation with malignant phenotype is the creation of a thick “coat” on the cell surface that hides cancer antigens and provides an escape of malignant cells from the immunosurveillance. Removing some sialic acid residues from the surface of malignant cells by sialidase can unmask cancer specific antigens and make cells visible to the immune system. The removal of sialic acids from tumor cells is associated with a reduced growth potential, an activation of NK cells, and a secretion of IFN-γ.^59^ The HN proteins present in SeV, NDV, and some other paramyxoviruses possess neuraminidase (sialidase) activities.^57,58^ Neuraminidase is capable of cleaving and removing sialic acid residues from the surface of malignant cells leading to a dramatic increase in their ability to induce the T-cell response.^60^

Some oncolytic viruses including paramyxoviruses are capable of inducing syncytia formation. During this process, infected and neighboring cells (up to 50 or even 100) are fusing with each other and are forming large multinucleated cells.^61^ We believe that the ability to trigger syncytium formation is another great advantage of oncolytic paramyxoviruses. Syncytium formation is a mechanism of spreading infection without the release of mature virus particles from cells. Most likely this mechanism contributes to the efficiency of viral oncolysis because it allows extra rounds of viral replication without any viral exposure to host neutralizing antibodies.

Moreover, syncytia are immunogenic formations; they secrete an abundance of “syncytiosomes,” which are exosome-like fluid filled cavities. The “syncytiosomes” present tumor-associated antigens (TAAs) through major histocompatability complex molecules.^62,63^ Death of syncytia is associated with autophagy.^64^ A number of paramyxoviruses, including MV,^65^ NDV,^66^ and SeV,^67^ have been shown to induce autophagic programmed cell death. It was demonstrated that autophagy within the antigen donor cells facilitates antigen cross-priming to generate TAA-specific or virus-specific CD8^+^ T cells, which could be further explored in the future as a strategy to enhance oncolytic viruses-mediated antitumor effects.^41^ In addition, cross-presentation of TAAs by DCs is strongly promoted by paramyxoviral fusogenic membrane glycoproteins.^63^

In summary, oncolytic paramyxoviruses are powerful anticancer immuno-stimulating agents. Strong anticancer effect of UV-inactivated viruses that cannot replicate or spread demonstrates most clearly the significance of the virus-induced anticancer immune response.^68,69^ Perhaps, isolated immune therapy with UV-inactivated viruses, which could not kill cancer cells directly through infection, should be most efficient in patients with relatively a small tumor burden. This approach is exploiting the UV-inactivated virus as tumor-debulking immunotherapy. It is likely that another approach of using alive and cancer cell replication competent virus strain could exploit both virus direct oncolysis and tumor debulking immunotherapy and perhaps could deal with larger tumor burden. It engages the array of mechanisms that can contribute to favorable therapeutic outcome and give hope to patients with the most advanced and surgically non removable cancers.

## Basic Properties of Paramyxoviruses

**Figure 1:**
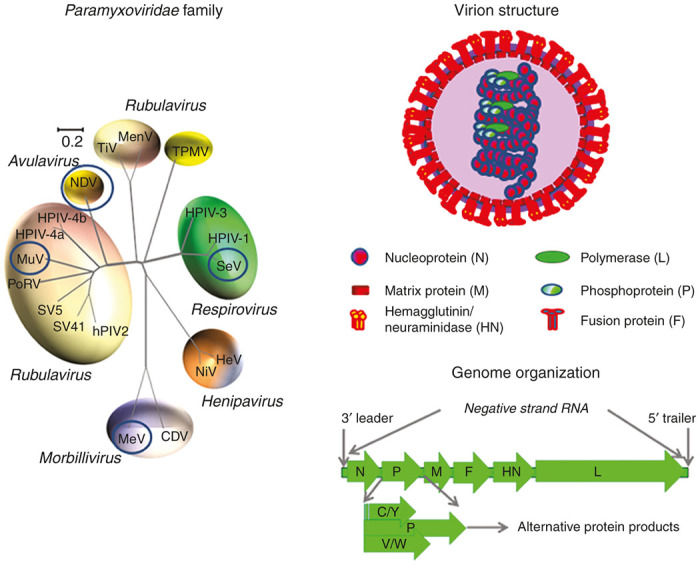
The *Paramyxoviridae* family. (**a**) The phylogenetic tree based on the alignment of the amino-acid sequences of the HN genes of selected *Paramyxoviridae* subfamily members. The virus representatives that demonstrated oncolytic properties are circled. The tree was generated from Clustal W multiple alignments^178^ using the neighbor-joining method.^179^ Viruses are grouped according to genus and abbreviated as follows. Morbillivirus genus: MV (Measles Virus), CDV (Canine Distemper Virus); Henipavirus genus: HeV (Hendra Virus), NiV (Nipah Virus); Respirovirus genus: SeV (Sendai Virus), HPIV1 (Human Parainfluenza Virus 1); HPIV3 (Human Parainfluenza Virus 3); Avulavirus genus: NDV (Newcastle Disease Virus); Rubulavirus genus: hPIV2 (Human Parainfluenza Virus 2), HPIV-4a (Human Parainfluenza Virus 4a), HPIV-4b (Human Parainfluenza Virus 4b), MuV (Mumps Virus), PoRV (Porcine Rubulavirus), SV5 (Simian Parainfluenza Virus 5), SV41 (Simian Parainfluenza Virus 41); TiV (Tioman Virus); MenV (Menangle Virus); Unclassified: TPMV (Tupaia Paramyxovirus). In 1993, the International Committee on the Taxonomy of Viruses re-classified the paramyxoviruses and placed NDV within the Rubulavirus genus. More recently, in 1999, a new genus, Avulavirus, has been created for the avian-specific Paramyxovirinae.^180^ This phylogenetic distinction is supported by comparative sequence analysis of RNA-dependent RNA polymerase proteins, matrix proteins and nucleocapsid proteins. The result of evolutionary comparative analysis of HN (hemagglutinin-neuraminidase) attachment proteins shown in this figure is in agreement with a classification that was suggested before 1999 revision. (**b**) Structure and composition of *Paramyxoviridae* virions. (**c**) Genome organization of paramyxoviruses and the minus strand RNA virus genome encoding genes.

Paramyxoviruses (members of the *Paramyxoviridae* family) are associated with a number of diseases in animals and humans, such as MV, mumps, and several respiratory infections (respiratory-syncytial virus (RSV), human parainfluenza viruses, metapneumovirus, etc.). Canine distemper virus (CDV) and Rinderpest virus are associated with lethal infections in dogs and cattle, respectively. Sendai virus (SeV) affects mice and some other animals. Newcastle disease virus (NDV) is associated with a contagious disease affecting many domestic and wild species of birds. Few representatives of paramyxoviruses NDV, SeV, MV, and mumps viruses were tested as oncolytic agents in multiple model experiments and in a few clinical trials. These representatives are marked with a circle in a phylogenetic tree of *Paramyxoviridae* family (Figure 1a).

Paramyxoviral virions are particles that are enveloped, spherical, or pleomorphic and 100–300 nm in diameter (Figure 1b). The nucleocapsid cores contain the genomic RNA covered by nucleocapsid proteins, in association with phosphoproteins (P) and RNA-dependent RNA polymerase proteins (RdRP or L). Matrix proteins inside the envelope stabilize the virus structure. Fusion proteins (F) and attachment proteins (H or HN or G) appear as spikes on the surface of the envelope. The attachment proteins could have hemagglutination activity (H protein), or combination of hemagglutinin with neuraminidase activity (HN-protein) that cleaves sialic acids from the cell surface. In some paramyxoviruses, the attachment glycoprotein (G) does not have these activities. All these proteins encoded by at least 6 genes in nonsegmented negative-sense single-stranded RNA genomes of ~15 kilobases (Figure 1c).

Paramyxoviruses enter the life cycle with binding of the attachment protein to an appropriate cell-surface receptor. There could be either direct fusion of the envelope with plasma membrane^70^ assisted by the F protein, which is activated by the interaction with sialic acid–containing surface glycoproteins,^18^ or the virus could enter the cell through the endocytic route where the fusion occurs in acidic conditions inside the endosomes.^71^ As result, the nucleocapsid containing viral genome is released into the cytosol where viral replication takes place.

The RdRP transcribes the genes into mRNAs, which are then translated into structural and nonstructural proteins. The transcription starts from a single promoter located at the 3′ end of the genome, and then may either terminate within specified regions between each viral gene, or proceed further downstream. Such mode of transcription is responsible for the observed product polarity in which the genes closest to the 3′ end of the genome are expressed more abundantly than their downstream counterparts.

The mechanism represents simple and effective way for transcription regulation that allows keeping viral products in the necessary balance. A concentration of the most abundantly synthesized nucleoprotein determines the moment when the RdRP switches from gene transcription to genome replication. The replication includes the synthesis of the full-length positive-strand RNAs, which are then transcribed into the progeny genomic minus-strand RNAs. The genomes associate with newly synthesized structural proteins forming nucleocapsids. The maturing virions finally gain their envelopes with the membrane-trapped viral glycoproteins by budding through the outer membrane. New virions can then infect other cells and enter new life cycles. An alternative pathway for spreading of the viral infection involves fusion of infected cells with their neighbors and formation of syncytia.^72^ Viral fusion proteins used by the virus to enter the cell are exposed to the cell surface of infected cells inducing fusion with plasma membranes of neighboring cells. Therefore, a single virion can potentially infect dozens of cells without any exposure to host neutralizing antibodies.

## Genomic Stability of Paramyxoviruses

In general, because of a lack of a replicative proofreading mechanism, RNA viruses have higher mutation rates than those of double-strand DNA viruses.^73^ However, negative-stranded RNA viruses (including paramyxoviruses) exhibit a low homologous recombination rate.^74,75^

Moreover, paramyxoviruses also belong to viruses that are governed by the “rule of six”, *i.e.*, their genomes mainly including six genes, which encode for six major proteins. The unusual genomic requirement for polyhexameric length (6n+0) is likely responsible for particular low rate of homologous RNA recombination in paramyxoviruses.^76^ This low rate probably contributes to comparatively high viral genomic stability. Other explanations for this relatively high genomic stability of paramyxoviruses also exist. Perhaps, tight cotranscriptional wrapping of a viral ribonucleoprotein complex of paramyxoviruses prevents homologous recombination.

Natural high genomic stability of paramyxoviruses is a positive feature for their potential use in any anticancer clinical application. For such application, it is important that viral and foreign genes would be expressed from a viral genome in a comparatively stable way, so many serial passages in cell cultures or embryonated chicken eggs can occur without genomic change.

## Preclinical Studies

### Sendai virus

The SeV virus is responsible for a highly transmissible respiratory tract infection in mice, hamsters, guinea pigs, and rats.^77^ It can be detected in animal colonies worldwide. While SeV spreads in rodents through both air and direct contact routes,^77^ it is considered quite safe for humans. Studies of the anticancer effects of SeV, which is also known as murine parainfluenza virus type 1 or hemagglutinating virus of Japan (HVJ), are mainly performed in Japan. SeV has oncolytic properties. Genetically engineered recombinant Sendai virus (rSeV) disseminates extensively in human tumor xenografts in nude mice without spreading to the surrounding normal cells.^78^ This dissemination leads to the inhibition of tumor growth in the mice. The tested tumor cells include fibrosarcoma, pancreatic epithelioid carcinoma, and human colon carcinoma.^78^ A significant reduction of tumor growth, including the complete elimination of established brain tumors, was demonstrated in murine models in a study using a different rSeV strain.^79^ Similar results were obtained with mouse xenografts of human sarcoma and prostate cancer.^78,80^ Recombinant SeV efficiently eliminated tumors in rat models, including melanoma, hepatocellular carcinoma, neuroblastoma, squamous cell carcinoma, and prostatic cancer.^81^ Some case studies that involved treatment of human cancers in a number of patients with SeV are reported in an issued patent^82^ and in patent application.^83^

Remarkably, the replication of SeV is not absolutely required for the anticancer effects of SeV as even UV-inactivated virus was shown to be efficient against colon,^68,69^ renal,^69^ and prostate carcinomas in mouse models.^52^ The UV-inactivated virus with enhanced antitumor activity was constructed by conjugation of IL-12 with hemagglutinin-neuraminidase (HN)-depleted viral particles (HVJ-E).^84^ It was demonstrated that this novel immune-stimulatory pseudovirion suppresses lung metastatic melanoma growth by regionally enhancing IFN-γ production without increasing the serum IFN-γ level.^84^

In all mentioned studies, SeV eradicated the tumors or significantly inhibited their growth. The apparent safety of the virus and the promising preclinical data suggest that SeV could be an excellent candidate for oncolytic virotherapy. Supporting these hopes is a case of a short-term remission in a patient with acute leukemia following an intravenous injection of live SeV described back in 1964.^85^

### Newcastle disease virus

Oncolytic properties of avian NDV, which belongs to the *Avulavirus* genus, have been studied for almost half of a century since the pioneering work of Flanagan *et al*.^86^ that was performed with mouse Ehrlich carcinoma. The availability of attenuated strains of NDV that are used as live vaccine for controlling the disease in the poultry industry provides additional opportunity of studying oncolytic effects with minimal hazard for the environment.^87^ NDV is well-studied oncolytic paramyxovirus,^88,89^ and the approach of using NDV for cancer therapy has already moved into phase 1, 2, and 3 clinical trials.

NDV strains have been classified into three categories according to their virulence and pathogenicity to avian species: velogenic (highly pathogenic), mesogenic (moderately pathogenic), and lentogenic (low pathogenic).^90^ NDV strains also have been also classified into two categories lytic and nonlytic according to their ability of infecting monolayer tumor cells. So, lytic strains in these cells produce infectious particles that can infect other tumor cells, thus leading to an amplification of the viral load, while non-lytic strains produce noninfectious particles. Besides, infection of lytic NDV strains results in syncytium formation. The NDV strains that have been evaluated for the treatment of human malignancies are the lytic mesogenic derived strains MTH68/H, PV-701, and 73-T and the nonlytic lentogenic derived strain Ulster and HUJ (Table 1).^90,91^

NDV was shown to kill human cancerous but not normal cells.^92,93^ A broad spectrum of human cancer cells were shown to be killed by NDV *in vitro*, which include tumor cell lines of ecto-, endo-, and mesodermal origin.^94^ Examples include the cells of colorectal, gastric, pancreatic, bladder, breast, ovarian, renal, lung, larynx, and cervical carcinomas, glioblastoma, melanoma,^95,96^ phaeochromocytoma,^97,98^ lymphomas of different origins,^95,99,100^ fibrosarcoma, osteosarcoma, neuroblastoma, and Wilms tumor.^92^ NDV has demonstrated a potent antitumor activity in several preclinical animal tumor models including neuroblastoma,^101^ fibrosarcoma,^102^ colon carcinomas,^103,104^ lung, breast, prostate carcinomas,^103^ hepatocellular carcinoma,^105^ and gastric carcinoma.^106^

In contrast, replication of NDV in nontumor cells could be up to 10,000-fold less efficient compared to some cancer cells,^95^ because in normal cells viral infection rapidly induces production of a number of antiviral proteins.^93^

### Measles virus

The anticancer effects of MV (genus *Morbillivirus*) have been studied for more than a decade.^107–109^ The number of publications describing anticancer effects of attenuated measles vaccine virus strains is rapidly growing. The Edmonston B strain is considered to be very safe, as it has lost its pathogenicity after extensive passages in tissue culture and in chicken embryos.^110^ This strain has shown potent antitumor activity against multiple primary as well as established tumor lines and in several preclinical animal tumor models, including both solid tumors and hematology malignancies.^61^ Examples include lymphoma,^111^ multiple myeloma,^112^ medulloblastoma,^113^ glioblastomamultiforme,^114^ hepatocellular carcinoma,^115^ prostate,^116^ breast,^117,118^ and ovarian cancer.^119^ Different routes of viral administration (intratumoral, intravenous, intraperitoneal, or intrapleural), and single- versus multiple-dose administration schemes have been used in these models.^61^ The characteristic cytopathic effect involving the formation of multinucleated cell aggregates was observed in the treated animal tumors, followed by the apoptotic death of the infected tumor cells. The studies with oncolytic strains of MV are now moved to clinical trials.

MV is a human pathogen and general public immunization exists against this virus. Is it a benefit or a problem for a perspective of using wide-scale MV constructs as oncolytic agents? On one hand, from a safety point of view, it is a benefit. On the another hand, from an oncolytic efficiency point of view it is a problem, because neutralizing antibodies against MV constructs could downplay therapeutic efficiency. So, the problem of preexisting immunity to MV virus should always be kept in mind in clinical trial designs.

Meantime, new recombinant variants with enhanced oncolytic activity are being engineered. One of the strategies is retargeting the virus to alternative receptors. Oncolytic MV can be fully genetically retargeted to specified cell surface receptors by elongating the attachment protein with designed ankyrin repeat protein.^120^ It can be also retargeted by modification of viral H-glycoprotein which is also responsible for interaction with cell receptors.^121^ The retargeting approach allows creating panels of oncolytic viruses with different targeting specificity, which is particularly useful for treating cancer cells that have lost expression of conventional receptors.

The ability to monitor viral gene expression *in vivo* in animal models and in patients represents an important challenge for oncolytic virotherapy. To meet this challenge, the construct MV that expresses carcinoembryonic antigen (MV-CEA) was created. Expression of soluble marker (CEA) by the construct provided an opportunity for rapid, cost efficient assessment of viral replication in any organism (model animal or human).

The convenience of viral replication monitoring through *in vivo* imaging inspired the creation and use of another MV construct that encoded human sodium-iodine symporter gene (NIS). The NIS gene, expressed from a viral construct in tumor cells, is capable of promoting accumulation of radioactive iodine isotopes in these cells. This accumulation allows imaging of a tumor’s localized viral gene expression. Moreover, the accumulation also promotes specific radio-damage of malignant cells, providing an opportunity for radiotherapy in addition to viral oncolytic and immuno-stimulating effects of MV therapy. Both MV constructs MV-CEA and MV-NIS demonstrated efficacy and safety in preclinical models (reviewed in refs. 122,123) and highly promising results in phase 1 clinical trials (see the section Measles Virus in Clinical Trials section).

The idea of virus oncolytic effect enhancement by other means (targeted chemotherapy) was further explored by creation of another viral construct. The MV construct with an inserted extra gene of the prodrug-converting enzyme for 5-FC was designed to transform the nontoxic compound into a highly cytotoxic drug. It was shown that the MV construct with such a suicide gene is able to infect, replicate, and kill malignant cells taken from cancer patients. It was also shown that addition of the prodrug to the cells significantly enhances the cell killing. The prodrug-converting enzyme was extensively expressed in MV-infected cells from the tumor slices of the patient-derived materials.^124^ A positive correlation was found between MV-infected tumor cell’s lysis and this cell’s overall incubation duration with the prodrug.^125^ In another study, the NCI-60 tumor cell panel was investigated for the cells’ susceptibility to a suicide gene-armed MV construct. It was found that ~50% of NCI-60 solid tumor cell lines are susceptible, ~40% are partially resistant and six tumor cell lines are highly resistance to the construct-induced oncolysis. The resistance was successfully overcome by addition of the prodrug 5-FC and increased multiplicity of the virus infection. Consequently, a successful prodrug activation approach for broadening the spectrum of malignant cells’ susceptibility to MV viral oncolysis was demonstrated.^126^

*In vivo*, intratumoral application of the MV suicide gene construct together with a systemic 5-FC treatment showed a significant reduction in tumor size in a xenograft mouse. The effect was stronger when compared with virus-only treatment.^127^ Intrahepatic and intraperitoneal applications of the same MV construct along with the systematic prodrug administration demonstrated comparative safety of the approach in animal models.^128^

## Clinical trials

A number of case studies and clinical trials (mainly phase 1 and 2) were performed with oncolytic paramyxoviruses. The results are summarized in Table 1 and outlined below.

### Sendai virus

### SeV (UV inactivated).

A phase 1/2a study for patients with advanced malignant melanoma was started recently in Osaka University (Japan).^129^

### SeV (alive).

SeV is a rodent pathogen. Administration of SeV to humans produces a very mild effect and does not cause any serious disease.^130^ Moreover, antibodies to SeV and to human Parainfluenza virus type 1 (hPIV-1) are cross-reactive,^130^ so it is highly likely that the majority of adult humans that naturally have antibodies to hPIV-1 also have preexisting immunity towards SeV.

According to personal communication of Dr Senin, SeV (Moscow strain) was tested in a few groups of patients with advanced metastatic cancers (Russia). Moscow trial included a group of 47 patients with disseminated advanced disease for whom surgeries were not safe to perform and SeV was used as a monotherapy. The primary tumor localizations were variable. Patients were treated with one or two cycles of viral therapy per year with each cycle representing 10^7^–10^8^ embryo infective dose 50% of SeV every 7–10 days during 4 months. Virus was injected intradermally and occasionally intratumorally along with specific pathogen free chicken embryos disintegrated cells that include cells that are permissive for SeV replication and infectious virions production. The embryo cells coinjection allowed extra cycles of viral replication in patients’ skin. The viral therapy caused mild flu-like symptoms and in general was well tolerated. In Moscow trial 31 out of 47 patients responded to therapy, with 6 major responses (complete primary tumor and metastases regression followed by 5–7 years of disease-free survival). St Petersburg trail included patients with advanced metastatic cancers after surgery. Whole cell vaccines prepared from patients cancer cells were added to SeV formulations described for Moscow trial. The rationale for this addition is that tumor-specific antigens that are generally located in the plasma membrane of cancer cells may be better recognized by the immune system if they are associated with virus antigens. The cancer cells are treated with gamma radiation to leave cells alive but prevent further cell division. The trial included two groups. The first group incorporated 15 patients with variable malignancies that enter the trial after debulking surgery. Four patients in this group responded to therapy. They experienced remaining tumor shrinking and survived at least 1 year without disease progression. The second group included 12 patients with variable malignancies that entered the trial after radical cytoreductive surgery. Eleven patients in this group responded to therapy, they were observed to be disease free for at least 1 year. Unfortunately, all patients from St Petersburg trail were lost for follow-up after only 1 year of observation. Some case studies that involved treatment of human cancers in a number of patients with SeV are reported in an issued patent^82^ and in a patent application.^83^

### Newcastle disease virus

NDV is considered to be low pathogenic for humans while being highly contagious and epizootic in many domestic and wild avian species.^131^ Administration of virulent strains of NDV to humans has been shown to result in only mild-to-moderate adverse effects, with mild conjunctivitis, laryngitis, and flu-like symptoms. Natural human infections with highly virulent avian NDV strains are possible, but they have been limited to mild conjunctivitis.^132^ The anticancer potential of NDV has been investigated in the United States, Canada, China, Germany, and Hungary. Several NDV strains (MTH-68/H, NDV-PV701, NDV-Ulster, and NDV-HUJ) have been the subject of systematic clinical studies in patients who had exhausted all conventional cancer treatments.

There are at least two different, but not mutually exclusive conceptual applications of NDV and other oncolytic viruses. In theory, oncolytic virus could be used for direct tumor selective oncolysis or it could be used for triggering immuno-mediated cancer killing. Oncolytic virus could be injected as a sole agent or as a component of a tumor vaccine. So, clinical studies have evaluated the use of NDV *per se*,^133–137^ or its combination with oncolysates as well as whole cell vaccines prepared from cancer cells (Table 1).^138–142^ The cancer cells could be used from autologous or allogenic material. Theoretically any of these approaches stimulate anticancer cytotoxic T cells (CTL) and other components of immune system, however, degree of this stimulation could be variable. Unfortunately, so far, clinical studies do not provide clear cut answers as to which approach is most beneficial.

NDV therapy was tested using different routes of delivery (intravenous, peritumoral, intratumoral, and others). The studies have shown that the viral therapy causes mild flu-like symptoms and in general is well tolerated. It is lacks toxicity even at very high doses applied systemically.^143,144^

NDV-based anticancer therapy has been reported to be of benefit in more than a dozen clinical trials. However, the majority of the trials were comparatively small with less than a few dozen patients per trial. The efficiency of treatment was demonstrated for melanomas, glyoblastomas, head and neck squamous cell carcinomas, and some other malignant diseases.

The mode of virus delivery seems to play a role in the efficiency of the cancer therapy. It was noted that intra- and peritumoral applications of NDV produce a stronger response compared to the systemic delivery, even when applied at much higher doses.^103,145^ Among larger trials, two have to be mentioned. A phase 1 clinical trial with more than 100 patients suffering from variable advanced malignancies was performed in a few locations in Canada and in the United States. NDV was injected intravenously^136,137,146,147^ and demonstrated safety at high viral doses. It was noticed that some objective responses to the therapy occurred only at the highest dose levels. A phase 3 trial that involved 50 colorectal cancer patients was completed in Germany. It studied the efficiency of active specific immunization with NDV infected autologous tumor cell vaccine, following resection of liver metastases. In the total patient group, no significant difference in the disease-free survival or overall survival was detected between treated and control categories. However, in the subgroup of colon cancer patients improved 10-year overall survival was observed due to NDV therapy (hazard ratio: 3.3; 95% confidence interval (CI): 1.0–10.4; *P* = 0.042).^142^

### Measles virus

The overview of the early experience in ongoing clinical trials studies of MV constructs with patients suffering with ovarian cancer, glioblastoma multiforme, multiple myeloma, and cutaneous T-cell lymphoma^122,148^ is highly encouraging. Heinzerling *et al*. performed first phase 1 clinical trial of MV antitumor virotherapy using the Edmonston–Zagreb strain of MV on patients with cutaneous T-cell lymphoma. The study showed that intratumoral injection of MV induced local infection and characteristic cytopathogenic effect of virus on tumor cells, which was not abrogated by the presence of preexisting MV antibodies. Tumor regressions occurred in three out of five patients. Interestingly, some regression of distant lesions where MV was not injected was observed.^149^

Addition of CEA antigen encoding sequence into the viral construct allowed cost efficient and quantitative *in vivo* monitoring of viral gene expression in tumors of ovarian cancer patients that are negative for the CEA cancer antigen. The monitoring demonstrated that, after intraperitoneal delivery, the construct was able to infect patients’ tumor cells. *De novo* expression of CEA by these tumor cells was observed.^150^

Other interesting observations from the MV-CEA study include: (i) Anti-measles antibody titers in blood and in peritoneal fluid remain at constant level following multiple viral intraperitoneal deliveries. So, in other words, despite repeated viral administration, antibody titer boosting effects were not observed. (ii) Measles infection with wild type virus frequently leads to transient but strong immuno-suppression. No evidence of MV-CEA construct treatment-induced immuno-suppression was observed. (iii) There was no evidence of virus shedding in patients’ mouth gargle or urine samples. However, in a few patients, viral genomes were detected at low levels in the peripheral blood mononuclear cells.^150^

Most importantly, intraperitoneal treatment with MV-CEA of patients suffering from recurrent ovarian cancer revealed dose-dependent disease stabilization in the majority of patients (14 of 21). No dose-limiting toxicity with intraperitoneal infusion of viral material with a dose escalation of up to 10^9^ tissue culture infective dose 50% was observed. The median survival time of the virus-treated patients (more than 12 months) was double that of the median survival of the nontreated patient population (6 months). Side effects were grade 1 and 2, with the most common among them being mild fever, fatigue, and mild abdominal pain.^150^

Addition of the human sodium-iodine symporter gene (NIS) into the MV construct, allowed *in vivo* imaging of viral replication in malignant cells. This imaging became possible due to the ability of the NIS gene product from the MV construct to promote accumulation of radioactive iodine isotopes in tumor cells. The approach of *in vivo* visualization of MV-NIS replication was tested in preclinical studies (reviewed in refs. 122,123) and in clinical trials (see below).

Two relapsing drug-refractory myeloma patients with low pretreatment serum titers of antimeasles antibodies were treated by MV-NIS construct. After only one i.v. viral infusion, tumor-selective replication was observed. This tumor-selective viral replication led to complete remission of a disseminated malignancy in one patient. The remission lasted at least 9 months. All toxicities were comparatively mild and resolved within the first week after therapy.^151^

Another clinical study with the same viral construct and ovarian cancer patients demonstrated that no dose limiting toxicity with intraperitoneal infusion of the viral material with up to 10^9^ tissue culture infectious dose 50% was observed. Treatment was associated with a median overall survival of 26.5 months, which compared favorably with outcomes of therapies without the viral construct.^152^

### Mumps virus

A few studies have been carried out with wild-type and attenuated mumps virus. The attenuated virus was tested in a group of 22 patients with advanced gynecologic malignancies,^153^ and wild type in a group of 90 patients with various malignancies at terminal stages^154^ as well as in a group of 200 patients with advanced cancers.^155^ The majority of the patients from these clinical trials experienced long-term suppression of tumor growth and certain oncolytic effects. For example, five of the seven patients with ascites experienced complete remission with no recurrence after intracavitary administration of the attenuated virus.^153^ Intravenous injections of the wild type mumps virus into 200 cancer patients caused a decrease or disappearance of ascites and edema of the lower limbs at high rates, a decrease or stoppage of cancerous bleeding and regression of tumors in 26 patients with cancer of the breast, rectum, thyroid gland, uterus, skin, etc.^155^ The published studies with mumps virus did not report median progression free or disease free survival times after therapy. Long-time survival benefit of treatment is unknown.

## Perspectives

Enhancing virus cancer cell killing abilities and diminishing bystander normal cells damage are modern day challenges for oncolytic virotherapy. These challenges trigger research that has already resulted in a number of interesting approaches that are waiting to be tested in clinical trials.

### Insertion of prodrug-converting enzyme

MV construct with an ability to transform the nontoxic compound into a highly cytotoxic drug (5-FC) was described above (see section Preclinical Trials, Measles Virus). So far, its efficiency was tested with the MV construct being administered locally. The ability of the construct to infect malignant cells *in vivo* in patients after systematic administration will define how broad its application is going to be.

### Insertion of GM-CSF–encoding gene

Another approach for improving antitumor virus therapy efficiency involves insertion of the GM-CSF encoding gene into an MV genome.^156^ In the colon adenocarcinoma model, it was demonstrated that the MV construct significantly delayed tumor progression and prolonged median overall survival of treated animals. The effect of intratumoral application of the GM-CSF gene encoding MV construct compared favorably with the effect of MV virus without the inserted gene. Evidence was obtained that arming MV with GM-CSF improves the attraction of immune cells to a tumor and enhances the induction of a tumor-specific immune response.^156^ It is highly likely that the encouraging results obtained using the murine model can be translated into successful clinical trials benefiting cancer patients.

### Combination with check-point inhibitors

The immunotherapeutic potential of oncolytic virus could be enhanced by coadministration of antibodies directed toward certain immuno-suppressive proteins. Two such proteins are of particular interest. One of them is cytotoxic T-lymphocyte-associated protein 4 (CTLA-4), which is a protein receptor that acts as an “off” switch when bound to some receptors on the surface of antigen presenting cells. Antibody to this receptor was already successfully clinically tested as an anticancer agent.^157^ Another is programmed death-ligand 1 (PD-L1), which is a protein that also suppresses the immune system. Antibody to this protein was also successfully clinically tested as an anticancer agent.^158^ Using animal models and poorly immunogenic tumors, it was demonstrated that antibodies towards (CTLA-4) act synergistically with oncolytic virus. Preestablished distant tumors were efficiently rejected after local intratumoral NDV administration along with systematic CTLA-4 antibody administration. The effect was independent of tumor cell line sensitivity to NDV-mediated lysis.^159^

Another study with animal models showed that CTLA-4 and PD-L1 checkpoint blockade enhances oncolytic effect of MV. The effect was demonstrated by using a MV construct with extra genes that encoded CTLA-4 and PD-L1 antibodies. It was confirmed by coadministration of MV vectors along with CTLA-4 and PD-L1 antibodies.^160^

### Improving oncotropism by decreasing off-target virus replication in normal cells

Differential expression of miRNAs in normal versus malignant cells can be explored to increase oncolytic virus specificity, which improves its safety after systematic administration. Synthetic target sites for miRNAs that are overexpressed in liver, gastrointestinal tract, and other organs were inserted into the MV genome. It was demonstrated that a replication of such MV construct was repressed in nontransformed primary human hepatocytes and in liver slices. So, the virus replication was affected in cells expressing relevant miRNAs.^161^ This work shows that expression levels of miRNAs in normal tissues can be used for adjusting virus oncotropism without compromising its oncolytic efficacy.

### Improving cancer cell–specific viral fusion protein (F_0_) processing

Paramyxoviral replication cycle requires protease cleavage of viral glycoproteins.^25^ For these viruses, a proteolytic processing enzyme is responsible for cleavage of precursor fusion glycoprotein F_0_ into fully active fusion protein dimer F_1_/F_2_. The functioning dimer protein is crucially important for the virions infectivity and syncytium formation. F_0_ is unable to fuse viral and cellular membranes for enabling viral penetration; moreover, it cannot direct fusion of infected cells with adjoining cells for syncytia formation. So, the malignant cell that does not express proteolytic processing enzymes either produces noninfectious defective virions or does not produce them at all.

The evidence exists that cancer specifically overexpressed proteinases could finalize the oncolytic cycle of paramyxoviruses. For example, MV virus requires proteinase-convertase furin for F_0_ processing, which is frequently overexpressed in metastatic cancers.^162^ Furin is a member the proprotein convertase (PC) family, processes inactive precursor proteins to functional proteins. Furin and other PC family members (furin/PCs) activate a number of proteins, including matrix metalloproteases (MMPs).^163^ MMP are specifically overexpressed in many metastatic cancers.^23,24^ The metastatic cells frequently secrete MMP, which is able to degrade the extracellular matrix and help these cells to metastasize. So, the expression and activity of furin as a proprotein convertase is necessary for processing of the enzymes, which stimulate tumor progression and metastasis.^163^ In summary, furin plays a significant role in tumor progression, invasiveness, and metastasis, so cancer cells frequently overexpress this protein.^164–168^ This furin overexpression most likely contributes to MV oncospecificity.

However, not all cancer cells express furin. So, the cancer cells that do not express this protein most likely are more resistant to MV oncolysis versus those cells that do express furin. Furin nonexpressing cells cannot produce infectious MV particles, which promote intratumoral syncytia formation. The introduction of the multi-basic cleavage site into the F-protein allows this protein to be cleaved and to be activated by a broader range of proteases. In place of a gene encoded furin-activated F-protein, a gene encoded MMP activated F-protein was inserted. The recombinant virus infection was spread only in cells secreting MMP. So, this recombinant MV obtained ability to infect, destroy, and spread in tumors expressing MMP.^162^ However, in so far as furin is activating MMP activity, it is not known if cancer cell’s MMP proteolytic activity is possible without furin being expressed.

To increase oncolytic potency of one lentogenic NDV strain, a polybasic cleavage site was also introduced into the F protein to generate a new site. While the resultant virus exhibited only an intermediate virulence phenotype based on a mean death time in embryonated eggs, the virus formed large syncytia and was enhanced in its replication in cancer cells, leading to enhanced oncolytic effects in various animal tumor models.^89,96,104,106,169–171^ Similar results were obtained when the F protein of the NDV La Sota strain was modified in an analogous fashion.^172,173^ “This improvement in syncytia formation caused a significant 20% prolongation of survival”.^89,174^

The studies with SeV also demonstrated that introduction of specific cleavage site into the F protein allows utilization of broad range of tumor tissue-specific proteases.^78,175,176^ Recombinant F protein of SeV enables its continuous spread in malignant human tissues without evidence of biodistribution into nonmalignant tissues.^177^

### Toward developing oncolytic virus sensitivity tests

A number of factors define how well a particular patient will respond to oncolytic virus therapy. Among them are the patient’s immune status and a sensitivity of his malignant cells to a particular viral infection. It is highly desirable to develop cost efficient approaches for analyzing this sensitivity without testing virus infections in primary cultures from biopsy or surgical material. Investigation of individual’s malignant cells for their potential susceptibility to oncolytic virus infection and ability to produce infectious virions has to be fast and cheap.

At least three factors contribute to development of permissive status by malignant cells that allows production of infectious paramyxovirus virions. First, a presence of a particular virus receptor, second, an absence of functioning IFN or/and apoptosis response pathways and, third, an expression of proteolytic processing enzymes for the F_0_–protein. Any malignant cell that is characterized by all three criteria mentioned above is more likely going to produce infectious virions and efficiently participate in intratumoral infection spread. The tumor, which consists of such cells, is likely vulnerable to oncolytic virus infection and consequent destruction.

So, an investigation of this vulnerability is needed to detect the category of the patients that are going to respond to the oncolytic therapy. Consequently, it is highly desirable to test the malignant cells from the patients’ biopsy or surgical material for the presence, quantity of viral receptors, for status of IFN response mechanism and for expression of the F_0_–protein processing proteolytic enzymes.

### General remarks about clinical trials and perspectives

Overall, clinical study results are still preliminary, and the reported outcomes are very diverse. So far, it is difficult to compare the relative efficiencies of the different paramyxoviruses in clinical trials. Median progression-free survival, median disease-free survival time as well as hazard ratios of experimental versus well-matched control groups are frequently not reported. However, the studies demonstrate comparative safety of oncolytic paramyxoviruses and, frequently, some objective positive effects. This information is encouraging for the design of phase 2/3 trials.

Future trials are likely going to be more successful because the factors that define how well a particular patient will respond to oncolytic virus therapy now are better understood. Consequently, better selection of the most likely responders among new patients becomes possible. Modern research demonstrated that the immunotherapeutic potential of oncolytic paramyxoviruses could be enhanced by coadministration of antibodies toward certain immuno-suppressive proteins. Moreover, the new sets of genetically modified viruses with enhanced virus cancer cell killing abilities as well as with optimized safety profiles are being made ready for clinical trials.

## Figures and Tables

**Table 1:** Clinical trials with inactivated and oncolytic paramyxoviruses

*Viral strain*	*Route of administration*	*Material, dosage, duration*	*Disease*	*Clinical study and patient number (n)*	*Clinical outcome*	*Year*	*References*
Sendai virus							
Sendai virus (UV inactivated VR105, *i.e.* Sendai/52)	Intratumoral	Viral particles, 6 times injection with 3,000 mNAU or 10,000 mNAU of inactivated SeV in 2 weeks/one cycle. Two cycles with 4-week interval	Advanced melanoma	Phase 1/2a (*n* = 6)	In progress	2009	Personal communication of Dr Kaneda
Sendai virus alive (Moscow strain)	Intradermal and/or intratumoral	Virus mixed with chicken embryo cells, 10^7^–10^8^ EID50 virus and 2 × 10^7^ cells, every 7–10 days during 4 months	Advanced cancers without metastases debulking	Case series (*n* = 47)	31 out of 47 patients responded to therapy, with six major responses (complete tumor regression followed by 5–7 years of disease-free survival)	1995	Personal communication of Dr V. Senin
		The same as above plus allogenic or autologous cells (0.5–2 × 10^7^ cells), every 7–10 days during 4 months	Advanced metastatic cancers after tumor debulking surgery	Case series (*n* = 15)	4 out of 15 patients experienced remaining tumor shrinking and survived at least 1 year without disease progression	1996	
	Intradermal		Advanced metastatic cancers after radical cytoreductive surgery	Case series (*n* = 12)	11 out of 12 patients experienced disease-free survival for at least 1 year	1996	
Newcastle disease virus							
Attenuated NDV							
NDV-73 T mesogenic	Subcutaneous injection	Allogenic or autologous human melanoma cells infected with NDV, weekly injections	Melanoma (stage II)	Phase 2 (*n* = 83)	10 years of survival was above 60% versus 6–33% of historic control, 15 years survival was 55% for NDV-treated patients	1992	138,139
NDV-MTH-68 mesogenic	Inhalation	4 × 10^3^ PFU (biweekly for 6 months)	Advanced cancers	Phase 2 (*n* = 33)	1-year survival for 22 out of 33 versus 4 out of 26 in control group; 2-year survival for 7 out of 33 versus 9 out of 26 in control group	1993	133
NDV-MTH-68 mesogenic	i.v. and inhalation	2 × 10^7^ to 2.5 × 10^8^ PFU (dosage step + maintenance dose) daily with alternate route of delivery	Glioblastoma multiforme	Case series (*n* = 14)	7 out of 14 responses; four major, 5–9-year survival or more	2004	134
NDV-HUJ lentogenic	i.v.	Dose escalation up to 55 × 10^9^ EID50	Glioblastoma multiforme	Phase 1 (*n* = 11)	One patient out of 11 achieved a complete response, all others had progressive disease	2006	135
NDV-PV701 mesogenic	i.v.	Dose escalation up to 1.20 × 10^11^ PFU	Advanced cancers	Phase 1 (*n* = 79)	Objective responses occurred at higher dose levels. Four out of 79 responses: two major, two minor, progression-free survival ranged from 4 to 31 months	2002	144
NDV-PV701 mesogenic	i.v.	Dose escalation up to 1.20 × 10^11^ PFU	Advanced cancers	Phase 1 (*n* = 16)	One patient experienced near-complete response with at least 1-year survival, four others from the treatment group had disease stabilization	2006	147
		1.2 × 10^11^ PFU	Advanced cancers	Phase 1 (*n* = 18)	Six out of 18 responses: one complete, three partial, and two minor; six patients with 2-year survival or more	2007	136,137
NDV Ulster lentogenic	Intradermal injections	Allogenic or autologous tumor cells infected with NDV, biweekly injections,5 total plus	Glioblastoma multiforme	Phase 1/2 (n = 23)	One patient was long term survivor versus none in a control group of 87 patients	2004	181
		Per vaccine, 1 × 10^7^ tumor cells were incubated for 1 hour with 64 HA units of the NDV					
			Head and neck squamous cell carcinomas	Phase 2 (*n* = 20)	5-year survival was 51%	2005	140,141
			Advanced colorectal cancer after resection of liver metastases	Phase 3 (*n* = 50) (randomized)	Subgroup of colon cancer patients analysis revealed a significant advantage for vaccinated colon cancer patients with respect to overall survival (hazard ratio: 3.3; 95% confidence interval (CI): 1.0–10.4; *P* = 0.042) and metastases-free survival (hazard ratio: 2.7; 95% CI: 1.0–7.4; *P* = 0.047)	2009	142
Measles virus							
Attenuated MV							
MV-EZ	Intratumoral	10^2^–10^3^ TCID_50_	Subcutaneous T-cell lymphoma	Phase 1 (*n* = 5)	One out of five complete regression of injected lesion, three out of five partial regression of injected lesions and two of these patients also experienced partial regression of distant lesions	2005	149
MV-CEA	Intraperitoneal	10^3^–10^9^ TCID_50_ (every 4 weeks for 6 months)	Ovarian cancer	Phase 1 (*n* = 21)	14 out of 21 responses: mean 1-year survival; one patient with 3.2-year survival	2010	150
MV-CEA	Intratumoral/excised tumor cavity		Glioblastoma multiforme	Phase 1	In progress		
MV-NIS	Intravenous		Multiple myeloma	Phase 1	In progress. After only one viral infusion, tumor-selective MV-NIS replication was observed that led to complete remission of a disseminated malignancy in one patient out of two infused.	2014	151
MV-NIS	Intraperitoneal	10^8^–10^9^ TCID_50_ (every 4 weeks for 6 months)	Ovarian cancer	Phase 1	Median overall survival of 26.5 months	2015	152
Mumps virus							
WT mumps virus							
Urabe strain	Topically, intravenously, or by inhalation	10^5^–10^7^ TCID_50_	Advanced cancers	Phase 2 (*n* = 90)	79 out of 90 patients responded to therapy and 37 out of 79 got significant or complete tumor regression	1974	154
Urabe strain		10^8^–10^9^ PFU	Advanced cancers	Phase 2 (*n* = 200)	26 out of 200 patients experienced tumor regression; majority of others experienced objective symptoms relief	1978	155
Attenuated mumps virus							
Attenuated Urabe strain	Subcutaneous presensitization intraperitoneal, intrathoracic and intratumoral	10^8^–10^9^ PFU	Gynecologic cancers	Phase 2 (*n* = 22)	Five out of seven patients with malignant ascites or pleural effusions experienced complete disappearance of disease manifestation; however, patients with large tumor masses did not respond. A clinical response was obtained in patients with ascites or pleural fluid who had received viral preimmunization	1988	153

CEA, carcinoembryonic antigen; EID50, 50% embryo infective dose; EZ, Edmonston–Zagreb; HUJ, Hebrew University in Jerusalem; MTH, mesogenic strain of Newcastle disease virus; MV, measles virus; NAU, neuraminidase unit; NDV, Newcastle disease virus; NIS, thyroidal sodium iodide symporter; PFU, plaque-forming units; TCID_50_, 50% tissue culture infectious dose; WT, wild-type.

## Box

- Loss of normal tissue architecture eliminates the important physical barriers that efficiently contain virus spreading in the organism. Cancer tumor vasculature is generally abnormal and leaky,^11^ which potentially favors virus spreading through the tumor mass.^12^
- The surface of a cancer cell often overexpresses certain transmembrane proteins that serve as receptors for viruses. Metastatic cancer cells usually overexpress sialic acid residues,^13^ CD46,^14,15^ and nectin 4,^16,17^ molecules that are located on their surface and can serve as receptors for oncolytic paramyxoviruses.^18–20^
- Cancer cells are commonly defective for type 1 IFN induction and response to IFN treatment.^21^
- It is likely that matrix metalloproteinases^22^ that are specifically overexpressed in many metastatic cancer cells^23,24^ could finalize oncolytic paramyxoviral replication cycle that requires protease cleavage of viral glycoproteins.^25^

## References

[bib1] Dock, G (1904). The influence of complicating diseases upon leukemia. Am J Med Sci 127: 563–592.

[bib2] De Pace, N (1912). Sulla scomparsa di un enorme cancro vegetante del collo dell’utero senza cura chirurgica. Ginecologia 9: 82–89.

[bib3] Levaditi, C, and Nicolau, S (1923). Vaccine et neoplasmes. Ann Inst Pasteur 37: 443–447.

[bib4] Farber, S and Diamond, LK (1948). Temporary remissions in acute leukemia in children produced by folic acid antagonist, 4-aminopteroyl-glutamic acid. N Engl J Med 238: 787–793. 18860765 10.1056/NEJM194806032382301

[bib5] Svejda, J (1950). [Viruses and tumors]. Lek List 5: 688–689. 14805199

[bib6] Moore, AE (1954). Effects of viruses on tumors. Annu Rev Microbiol 8: 393–410. 13198114 10.1146/annurev.mi.08.100154.002141

[bib7] Kelly, E and Russell, SJ (2007). History of oncolytic viruses: genesis to genetic engineering. Mol Ther 15: 651–659. 17299401 10.1038/sj.mt.6300108

[bib8] Karapanagiotou, EM, Roulstone, V, Twigger, K, Ball, M, Tanay, M, Nutting, C et al. (2012). Phase I/II trial of carboplatin and paclitaxel chemotherapy in combination with intravenous oncolytic reovirus in patients with advanced malignancies. Clin Cancer Res 18: 2080–2089. 22316603 10.1158/1078-0432.CCR-11-2181 PMC5553618

[bib9] Heo, J, Reid, T, Ruo, L, Breitbach, CJ, Rose, S, Bloomston, M et al. (2013). Randomized dose-finding clinical trial of oncolytic immunotherapeutic vaccinia JX-594 in liver cancer. Nat Med 19: 329–336. 23396206 10.1038/nm.3089 PMC4268543

[bib10] Dolgin, E (2015). Oncolytic viruses get a boost with first FDA-approval recommendation. Nat Rev Drug Discov 14: 369–371. 26027526 10.1038/nrd4643

[bib11] De Bock, K, Cauwenberghs, S and Carmeliet, P (2011). Vessel abnormalization: another hallmark of cancer? Molecular mechanisms and therapeutic implications. Curr Opin Genet Dev 21: 73–79. 21106363 10.1016/j.gde.2010.10.008

[bib12] Ogris, M (2010). Nucleic acid therapeutics: concepts for targeted delivery to solid tumors. Ther Deliv 1: 91–107. 22816123 10.4155/tde.10.9

[bib13] Büll, C, den Brok, MH and Adema, GJ (2014). Sweet escape: sialic acids in tumor immune evasion. Biochim Biophys Acta 1846: 238–246. 25026312 10.1016/j.bbcan.2014.07.005

[bib14] Surowiak, P, Materna, V, Maciejczyk, A, Kaplenko, I, Spaczynski, M, Dietel, M et al. (2006). CD46 expression is indicative of shorter revival-free survival for ovarian cancer patients. Anticancer Res 26(6C): 4943–4948. 17214367

[bib15] Maciejczyk, A, Szelachowska, J, Szynglarewicz, B, Szulc, R, Szulc, A, Wysocka, T et al. (2011). CD46 Expression is an unfavorable prognostic factor in breast cancer cases. Appl Immunohistochem Mol Morphol 19: 540–546. 21617523 10.1097/PAI.0b013e31821a0be9

[bib16] Takano, A, Ishikawa, N, Nishino, R, Masuda, K, Yasui, W, Inai, K et al. (2009). Identification of nectin-4 oncoprotein as a diagnostic and therapeutic target for lung cancer. Cancer Res 69: 6694–6703. 19679554 10.1158/0008-5472.CAN-09-0016

[bib17] Derycke, MS, Pambuccian, SE, Gilks, CB, Kalloger, SE, Ghidouche, A, Lopez, M et al. (2010). Nectin 4 overexpression in ovarian cancer tissues and serum: potential role as a serum biomarker. Am J Clin Pathol 134: 835–845. 20959669 10.1309/AJCPGXK0FR4MHIHB PMC3042138

[bib18] Villar, E and Barroso, IM (2006). Role of sialic acid-containing molecules in paramyxovirus entry into the host cell: a minireview. Glycoconj J 23: 5–17. 16575518 10.1007/s10719-006-5433-0

[bib19] Noyce, RS, Bondre, DG, Ha, MN, Lin, LT, Sisson, G, Tsao, MS et al. (2011). Tumor cell marker PVRL4 (nectin 4) is an epithelial cell receptor for measles virus. PLoS Pathog 7: e1002240. 21901103 10.1371/journal.ppat.1002240 PMC3161989

[bib20] Noyce, RS and Richardson, CD (2012). Nectin 4 is the epithelial cell receptor for measles virus. Trends Microbiol 20: 429–439. 22721863 10.1016/j.tim.2012.05.006

[bib21] Haus, O (2000). The genes of interferons and interferon-related factors: localization and relationships with chromosome aberrations in cancer. Arch Immunol Ther Exp (Warsz) 48: 95–100. 10807049

[bib22] Cattaneo, R, Miest, T, Shashkova, EV and Barry, MA (2008). Reprogrammed viruses as cancer therapeutics: targeted, armed and shielded. Nat Rev Microbiol 6: 529–540. 18552863 10.1038/nrmicro1927 PMC3947522

[bib23] Egeblad, M and Werb, Z (2002). New functions for the matrix metalloproteinases in cancer progression. Nat Rev Cancer 2: 161–174. 11990853 10.1038/nrc745

[bib24] Hadler-Olsen, E, Winberg, JO and Uhlin-Hansen, L (2013). Matrix metalloproteinases in cancer: their value as diagnostic and prognostic markers and therapeutic targets. Tumour Biol 34: 2041–2051. 23681802 10.1007/s13277-013-0842-8

[bib25] Cattaneo, R (2010). Paramyxovirus entry and targeted vectors for cancer therapy. PLoS Pathog 6: e1000973. 20585633 10.1371/journal.ppat.1000973 PMC2891830

[bib26] Bian, T, Gibbs, JD, Örvell, C and Imani, F (2012). Respiratory syncytial virus matrix protein induces lung epithelial cell cycle arrest through a p53 dependent pathway. PLoS One 7: e38052. 22662266 10.1371/journal.pone.0038052 PMC3360651

[bib27] Muñoz-Fontela, C, Macip, S, Martínez-Sobrido, L, Brown, L, Ashour, J, García-Sastre, A et al. (2008). Transcriptional role of p53 in interferon-mediated antiviral immunity. J Exp Med 205: 1929–1938. 18663127 10.1084/jem.20080383 PMC2525597

[bib28] Rivas, C, Aaronson, SA and Munoz-Fontela, C (2010). Dual Role of p53 in Innate Antiviral Immunity. Viruses 2: 298–313. 21994612 10.3390/v2010298 PMC3185551

[bib29] Takaoka, A, Hayakawa, S, Yanai, H, Stoiber, D, Negishi, H, Kikuchi, H et al. (2003). Integration of interferon-alpha/beta signalling to p53 responses in tumour suppression and antiviral defence. Nature 424: 516–523. 12872134 10.1038/nature01850

[bib30] Zheltukhin, AO and Chumakov, PM (2010). Constitutive and induced functions of the p53 gene. Biochemistry (Mosc) 75: 1692–1721. 21418001 10.1134/s0006297910130110

[bib31] Chumakov, PM (2007). Versatile functions of p53 protein in multicellular organisms. Biochemistry (Mosc) 72: 1399–1421. 18282133 10.1134/s0006297907130019 PMC2709848

[bib32] Levine, AJ and Oren, M (2009). The first 30 years of p53: growing ever more complex. Nat Rev Cancer 9: 749–758. 19776744 10.1038/nrc2723 PMC2771725

[bib33] Tanaka, N, Ishihara, M, Lamphier, MS, Nozawa, H, Matsuyama, T, Mak, TW et al. (1996). Cooperation of the tumour suppressors IRF-1 and p53 in response to DNA damage. Nature 382: 816–818. 8752276 10.1038/382816a0

[bib34] Moiseeva, O, Mallette, FA, Mukhopadhyay, UK, Moores, A and Ferbeyre, G (2006). DNA damage signaling and p53-dependent senescence after prolonged beta-interferon stimulation. Mol Biol Cell 17: 1583–1592. 16436515 10.1091/mbc.E05-09-0858 PMC1415317

[bib35] Leonova, KI, Brodsky, L, Lipchick, B, Pal, M, Novototskaya, L, Chenchik, AA et al. (2013). p53 cooperates with DNA methylation and a suicidal interferon response to maintain epigenetic silencing of repeats and noncoding RNAs. Proc Natl Acad Sci USA 110: E89–E98. 23236145 10.1073/pnas.1216922110 PMC3538199

[bib36] Dunn, GP, Koebel, CM and Schreiber, RD (2006). Interferons, immunity and cancer immunoediting. Nat Rev Immunol 6: 836–848. 17063185 10.1038/nri1961

[bib37] Bartee, E and McFadden, G (2009). Human cancer cells have specifically lost the ability to induce the synergistic state caused by tumor necrosis factor plus interferon-beta. Cytokine 47: 199–205. 19640730 10.1016/j.cyto.2009.06.006 PMC4376283

[bib38] Katsoulidis, E, Kaur, S, and Platanias, LC (2010). Deregulation of interferon signaling in malignant cells. Pharmaceuticals 3 406–418. 10.3390/ph3020406 PMC4033917 27713259

[bib39] Hanahan, D and Weinberg, RA (2011). Hallmarks of cancer: the next generation. Cell 144: 646–674. 21376230 10.1016/j.cell.2011.02.013

[bib40] Berchtold, S, Lampe, J, Weiland, T, Smirnow, I, Schleicher, S, Handgretinger, R et al. (2013). Innate immune defense defines susceptibility of sarcoma cells to measles vaccine virus based oncolysis. J Virol 9: 9. 10.1128/JVI.02106-12 PMC3592150 23302892

[bib41] Bartlett, DL, Liu, Z, Sathaiah, M, Ravindranathan, R, Guo, Z, He, Y et al. (2013). Oncolytic viruses as therapeutic cancer vaccines. Mol Cancer 12: 103. 24020520 10.1186/1476-4598-12-103 PMC3847443

[bib42] Boisgerault, N, Tangy, F and Gregoire, M (2010). New perspectives in cancer virotherapy: bringing the immune system into play. Immunotherapy 2: 185–199. 20635927 10.2217/imt.10.6

[bib43] Prestwich, RJ, Errington, F, Diaz, RM, Pandha, HS, Harrington, KJ, Melcher, AA et al. (2009). The case of oncolytic viruses versus the immune system: waiting on the judgment of Solomon. Hum Gene Ther 20: 1119–1132. 19630549 10.1089/hum.2009.135 PMC2829276

[bib44] Donnelly, OG, Errington-Mais, F, Steele, L, Hadac, E, Jennings, V, Scott, K et al. (2013). Measles virus causes immunogenic cell death in human melanoma. Gene Ther 20: 7–15. 22170342 10.1038/gt.2011.205 PMC3378495

[bib45] Sze, DY, Reid, TR and Rose, SC (2013). Oncolytic virotherapy. J Vasc Interv Radiol 24: 1115–1122. 23885911 10.1016/j.jvir.2013.05.040

[bib46] Tai, LH, Zhang, J, Scott, KJ, de Souza, CT, Alkayyal, AA, Ananth, AA et al. (2013). Perioperative influenza vaccination reduces postoperative metastatic disease by reversing surgery-induced dysfunction in natural killer cells. Clin Cancer Res 19: 5104–5115. 23881927 10.1158/1078-0432.CCR-13-0246

[bib47] Yu, YA, Shabahang, S, Timiryasova, TM, Zhang, Q, Beltz, R, Gentschev, I et al. (2004). Visualization of tumors and metastases in live animals with bacteria and vaccinia virus encoding light-emitting proteins. Nat Biotechnol 22: 313–320. 14990953 10.1038/nbt937

[bib48] Lemay, CG, Rintoul, JL, Kus, A, Paterson, JM, Garcia, V, Falls, TJ et al. (2012). Harnessing oncolytic virus-mediated antitumor immunity in an infected cell vaccine. Mol Ther 20: 1791–1799. 22760544 10.1038/mt.2012.128 PMC3437573

[bib49] Bossart, KN, Fusco, DL and Broder, CC (2013). Paramyxovirus entry. Adv Exp Med Biol 790: 95–127. 23884588 10.1007/978-1-4614-7651-1_6 PMC8782154

[bib50] Matrosovich, M, Herrler, G, and Klenk, HD (2013). Sialic acid receptors of viruses. Top Curr Chem 7: 73. 10.1007/128_2013_466 PMC7120183 23873408

[bib51] Büll, C, Stoel, MA, den Brok, MH and Adema, GJ (2014). Sialic acids sweeten a tumor’s life. Cancer Res 74: 3199–3204. 24830719 10.1158/0008-5472.CAN-14-0728

[bib52] Kawaguchi, Y, Miyamoto, Y, Inoue, T and Kaneda, Y (2009). Efficient eradication of hormone-resistant human prostate cancers by inactivated Sendai virus particle. Int J Cancer 124: 2478–2487. 19173282 10.1002/ijc.24234

[bib53] Myers, R, Greiner, S, Harvey, M, Soeffker, D, Frenzke, M, Abraham, K et al. (2005). Oncolytic activities of approved mumps and measles vaccines for therapy of ovarian cancer. Cancer Gene Ther 12: 593–599. 15746945 10.1038/sj.cgt.7700823

[bib54] Anderson, BD, Nakamura, T, Russell, SJ and Peng, KW (2004). High CD46 receptor density determines preferential killing of tumor cells by oncolytic measles virus. Cancer Res 64: 4919–4926. 15256464 10.1158/0008-5472.CAN-04-0884

[bib55] Fabre-Lafay, S, Monville, F, Garrido-Urbani, S, Berruyer-Pouyet, C, Ginestier, C, Reymond, N et al. (2007). Nectin-4 is a new histological and serological tumor associated marker for breast cancer. BMC Cancer 7: 73. 17474988 10.1186/1471-2407-7-73 PMC1868744

[bib56] Matveeva, OV, Guo, ZS, Shabalina, SA, and Chumakov, PM (2015). Oncolysis by paramyxoviruses: multiple mechanisms contribute to therapeutic efficiency. Molecular Therapy — Oncolytics 2, 15011; doi:10.1038/mto.2015.11. 26640816 10.1038/mto.2015.11 PMC4667958

[bib57] Kingsbury, DW (1991). The Paramyxoviruses. Plenum Press: New York.

[bib58] Enders, G (1996). Paramyxoviruses. University of Texas Medical Branch at Galveston: Galveston. 21413341

[bib59] Cohen, M, Elkabets, M, Perlmutter, M, Porgador, A, Voronov, E, Apte, RN et al. (2010). Sialylation of 3-methylcholanthrene-induced fibrosarcoma determines antitumor immune responses during immunoediting. J Immunol 185: 5869–5878. 20956342 10.4049/jimmunol.1001635

[bib60] Powell, LD, Whiteheart, SW and Hart, GW (1987). Cell surface sialic acid influences tumor cell recognition in the mixed lymphocyte reaction. J Immunol 139: 262–270. 2953814

[bib61] Galanis, E (2010). Therapeutic potential of oncolytic measles virus: promises and challenges. Clin Pharmacol Ther 88: 620–625. 20881957 10.1038/clpt.2010.211

[bib62] Bateman, A, Bullough, F, Murphy, S, Emiliusen, L, Lavillette, D, Cosset, FL et al. (2000). Fusogenic membrane glycoproteins as a novel class of genes for the local and immune-mediated control of tumor growth. Cancer Res 60: 1492–1497. 10749110

[bib63] Bateman, AR, Harrington, KJ, Kottke, T, Ahmed, A, Melcher, AA, Gough, MJ et al. (2002). Viral fusogenic membrane glycoproteins kill solid tumor cells by nonapoptotic mechanisms that promote cross presentation of tumor antigens by dendritic cells. Cancer Res 62: 6566–6578. 12438252

[bib64] Delpeut, S, Rudd, PA, Labonté, P and von Messling, V (2012). Membrane fusion-mediated autophagy induction enhances morbillivirus cell-to-cell spread. J Virol 86: 8527–8535. 22647692 10.1128/JVI.00807-12 PMC3421762

[bib65] Richetta, C, Grégoire, IP, Verlhac, P, Azocar, O, Baguet, J, Flacher, M et al. (2013). Sustained autophagy contributes to measles virus infectivity. PLoS Pathog 9: e1003599. 24086130 10.1371/journal.ppat.1003599 PMC3784470

[bib66] Meng, C, Qiu, X, Jin, S, Yu, S, Chen, H and Ding, C (2012). Whole genome sequencing and biological characterization of Duck/JS/10, a new lentogenic class I Newcastle disease virus. Arch Virol 157: 869–880. 22310996 10.1007/s00705-012-1248-4

[bib67] Siddiqui, MA and Malathi, K (2012). RNase L induces autophagy via c-Jun N-terminal kinase and double-stranded RNA-dependent protein kinase signaling pathways. J Biol Chem 287: 43651–43664. 23109342 10.1074/jbc.M112.399964 PMC3527951

[bib68] Kurooka, M and Kaneda, Y (2007). Inactivated Sendai virus particles eradicate tumors by inducing immune responses through blocking regulatory T cells. Cancer Res 67: 227–236. 17210703 10.1158/0008-5472.CAN-06-1615

[bib69] Fujihara, A, Kurooka, M, Miki, T and Kaneda, Y (2008). Intratumoral injection of inactivated Sendai virus particles elicits strong antitumor activity by enhancing local CXCL10 expression and systemic NK cell activation. Cancer Immunol Immunother 57: 73–84. 17602226 10.1007/s00262-007-0351-y PMC11030187

[bib70] Lamb, RA, and Parks, GD (2007). Paramyxoviridae: the viruses and their replication. In: Knipe, DM, and Howley, PM (eds). Fields Virology, 5th edn. Lippincott Williams & Wilkins: Philadelphia, pp. 1449–1496.

[bib71] Cantín, C, Holguera, J, Ferreira, L, Villar, E and Muñoz-Barroso, I (2007). Newcastle disease virus may enter cells by caveolae-mediated endocytosis. J Gen Virol 88(Pt 2): 559–569. 17251575 10.1099/vir.0.82150-0

[bib72] Higuchi, H, Bronk, SF, Bateman, A, Harrington, K, Vile, RG and Gores, GJ (2000). Viral fusogenic membrane glycoprotein expression causes syncytia formation with bioenergetic cell death: implications for gene therapy. Cancer Res 60: 6396–6402. 11103804

[bib73] Elena, SF, Bedhomme, S, Carrasco, P, Cuevas, JM, de la Iglesia, F, Lafforgue, G et al. (2011). The evolutionary genetics of emerging plant RNA viruses. Mol Plant Microbe Interact 24: 287–293. 21294624 10.1094/MPMI-09-10-0214

[bib74] Chare, ER, Gould, EA and Holmes, EC (2003). Phylogenetic analysis reveals a low rate of homologous recombination in negative-sense RNA viruses. J Gen Virol 84(Pt 10): 2691–2703. 13679603 10.1099/vir.0.19277-0

[bib75] Han, GZ and Worobey, M (2011). Homologous recombination in negative sense RNA viruses. Viruses 3: 1358–1373. 21994784 10.3390/v3081358 PMC3185808

[bib76] Kolakofsky, D, Roux, L, Garcin, D and Ruigrok, RW (2005). Paramyxovirus mRNA editing, the “rule of six” and error catastrophe: a hypothesis. J Gen Virol 86(Pt 7): 1869–1877. 15958664 10.1099/vir.0.80986-0

[bib77] Institute of Laboratory Animal Resources (U.S.). Committee on Infectious Diseases of Mice and Rats. (1991). Infectious Diseases of Mice and Rats. National Academy Press: Washington, DC.

[bib78] Kinoh, H and Inoue, M (2008). New cancer therapy using genetically-engineered oncolytic Sendai virus vector. Front Biosci 13: 2327–2334. 17981715 10.2741/2847

[bib79] Iwadate, Y, Inoue, M, Saegusa, T, Tokusumi, Y, Kinoh, H, Hasegawa, M et al. (2005). Recombinant Sendai virus vector induces complete remission of established brain tumors through efficient interleukin-2 gene transfer in vaccinated rats. Clin Cancer Res 11: 3821–3827. 15897582 10.1158/1078-0432.CCR-04-1485

[bib80] Tatsuta, K, Tanaka, S, Tajiri, T, Shibata, S, Komaru, A, Ueda, Y et al. (2009). Complete elimination of established neuroblastoma by synergistic action of gamma-irradiation and DCs treated with rSeV expressing interferon-beta gene. Gene Ther 16: 240–251. 18987675 10.1038/gt.2008.161

[bib81] Yonemitsu, Y, Ueda, Y, Kinoh, H and Hasegawa, M (2008). Immunostimulatory virotherapy using recombinant Sendai virus as a new cancer therapeutic regimen. Front Biosci 13: 1892–1898. 17981677 10.2741/2809

[bib82] Senin, V, Senina, A, and Matveeva, O (2014). Method for cancer immunotherapy and pharmaceutical compositions based on oncolytic non-pathogenic Sendai virus. Russian Federation, patent N 2519763, Russia.

[bib83] Senin, V, Senina, A, and Matveeva, O (2014). Method for cancer immunotherapy and pharmaceutical compositions based on oncolytic non-pathogenic Sendai virus. Patent application number PCT/RU2013/001043 and publication number WO2014081346 A3.

[bib84] Saga, K, Tamai, K, Yamazaki, T and Kaneda, Y (2013). Systemic administration of a novel immune-stimulatory pseudovirion suppresses lung metastatic melanoma by regionally enhancing IFN-γ production. Clin Cancer Res 19: 668–679. 23251005 10.1158/1078-0432.CCR-12-1947

[bib85] Wheelock, EF and Dingle, JH (1964). Observations on the repeated administration of viruses to a patient with acute leukemia. A preliminary report. N Engl J Med 271: 645–651. 14170843 10.1056/NEJM196409242711302

[bib86] Flanagan, AD, Love, R and Tesar, W (1955). Propagation of Newcastle disease virus in Ehrlich ascites cells *in vitro* and *in vivo*. Proc Soc Exp Biol Med 90: 82–86. 13273360 10.3181/00379727-90-21945

[bib87] Lech, PJ and Russell, SJ (2010). Use of attenuated paramyxoviruses for cancer therapy. Expert Rev Vaccines 9: 1275–1302. 21087107 10.1586/erv.10.124

[bib88] Fournier, P, Bian, H, Szeberényi, J and Schirrmacher, V (2012). Analysis of three properties of Newcastle disease virus for fighting cancer: tumor-selective replication, antitumor cytotoxicity, and immunostimulation. Methods Mol Biol 797: 177–204. 21948477 10.1007/978-1-61779-340-0_13

[bib89] Zamarin, D and Palese, P (2012). Oncolytic Newcastle disease virus for cancer therapy: old challenges and new directions. Future Microbiol 7: 347–367. 22393889 10.2217/fmb.12.4 PMC4241685

[bib90] Lam, HY, Yeap, SK, Rasoli, M, Omar, AR, Yusoff, K, Suraini, AA et al. (2011). Safety and clinical usage of Newcastle disease virus in cancer therapy. J Biomed Biotechnol 718710: 26. 10.1155/2011/718710 PMC3205905 22131816

[bib91] Csatary, LK and Csatary, CM (2013). Clone of Newcastle disease virus, its manufacture and its application in the medical treatment of cancer. US patent 8377450 B2. United Cancer Research Institute: USA.

[bib92] Reichard, KW, Lorence, RM, Cascino, CJ, Peeples, ME, Walter, RJ, Fernando, MB et al. (1992). Newcastle disease virus selectively kills human tumor cells. J Surg Res 52: 448–453. 1619912 10.1016/0022-4804(92)90310-v

[bib93] Fiola, C, Peeters, B, Fournier, P, Arnold, A, Bucur, M and Schirrmacher, V (2006). Tumor selective replication of Newcastle disease virus: association with defects of tumor cells in antiviral defence. Int J Cancer 119: 328–338. 16470838 10.1002/ijc.21821

[bib94] Fábián, Z, Csatary, CM, Szeberényi, J and Csatary, LK (2007). p53-independent endoplasmic reticulum stress-mediated cytotoxicity of a Newcastle disease virus strain in tumor cell lines. J Virol 81: 2817–2830. 17215292 10.1128/JVI.02490-06 PMC1865991

[bib95] Schirrmacher, V, Haas, C, Bonifer, R, Ahlert, T, Gerhards, R and Ertel, C (1999). Human tumor cell modification by virus infection: an efficient and safe way to produce cancer vaccine with pleiotropic immune stimulatory properties when using Newcastle disease virus. Gene Ther 6: 63–73. 10341877 10.1038/sj.gt.3300787

[bib96] Zamarin, D, Martínez-Sobrido, L, Kelly, K, Mansour, M, Sheng, G, Vigil, A et al. (2009). Enhancement of oncolytic properties of recombinant newcastle disease virus through antagonism of cellular innate immune responses. Mol Ther 17: 697–706. 19209145 10.1038/mt.2008.286 PMC2835121

[bib97] Fábián, Z, Töröcsik, B, Kiss, K, Csatary, LK, Bodey, B, Tigyi, J et al. (2001). Induction of apoptosis by a Newcastle disease virus vaccine (MTH-68/H) in PC12 rat phaeochromocytoma cells. Anticancer Res 21: 125–135. 11299726

[bib98] Szeberényi, J, Fábián, Z, Töröcsik, B, Kiss, K and Csatary, LK (2003). Newcastle disease virus-induced apoptosis in PC12 pheochromocytoma cells. Am J Ther 10: 282–288. 12845392 10.1097/00045391-200307000-00008

[bib99] Tzadok-David, Y, Metzkin-Eizenberg, M and Zakay-Rones, Z (1995). The effect of a mesogenic and a lentogenic Newcastle disease virus strain on Burkitt lymphoma Daudi cells. J Cancer Res Clin Oncol 121: 169–174. 7713989 10.1007/BF01198099 PMC12201484

[bib100] Bar-Eli, N, Giloh, H, Schlesinger, M and Zakay-Rones, Z (1996). Preferential cytotoxic effect of Newcastle disease virus on lymphoma cells. J Cancer Res Clin Oncol 122: 409–415. 8690751 10.1007/BF01212880 PMC12201453

[bib101] Lorence, RM, Reichard, KW, Katubig, BB, Reyes, HM, Phuangsab, A, Mitchell, BR et al. (1994). Complete regression of human neuroblastoma xenografts in athymic mice after local Newcastle disease virus therapy. J Natl Cancer Inst 86: 1228–1233. 8040891 10.1093/jnci/86.16.1228

[bib102] Lorence, RM, Katubig, BB, Reichard, KW, Reyes, HM, Phuangsab, A, Sassetti, MD et al. (1994). Complete regression of human fibrosarcoma xenografts after local Newcastle disease virus therapy. Cancer Res 54: 6017–6021. 7954437

[bib103] Phuangsab, A, Lorence, RM, Reichard, KW, Peeples, ME and Walter, RJ (2001). Newcastle disease virus therapy of human tumor xenografts: antitumor effects of local or systemic administration. Cancer Lett 172: 27–36. 11595126 10.1016/s0304-3835(01)00617-6

[bib104] Vigil, A, Park, MS, Martinez, O, Chua, MA, Xiao, S, Cros, JF et al. (2007). Use of reverse genetics to enhance the oncolytic properties of Newcastle disease virus. Cancer Res 67: 8285–8292. 17804743 10.1158/0008-5472.CAN-07-1025

[bib105] Altomonte, J, Marozin, S, Schmid, RM and Ebert, O (2010). Engineered newcastle disease virus as an improved oncolytic agent against hepatocellular carcinoma. Mol Ther 18: 275–284. 19809404 10.1038/mt.2009.231 PMC2839313

[bib106] Song, KY, Wong, J, Gonzalez, L, Sheng, G, Zamarin, D and Fong, Y (2010). Antitumor efficacy of viral therapy using genetically engineered Newcastle disease virus [NDV(F3aa)-GFP] for peritoneally disseminated gastric cancer. J Mol Med (Berl) 88: 589–596. 20393691 10.1007/s00109-010-0605-6 PMC3269811

[bib107] Nakamura, T and Russell, SJ (2004). Oncolytic measles viruses for cancer therapy. Expert Opin Biol Ther 4: 1685–1692. 15461580 10.1517/14712598.4.10.1685

[bib108] Blechacz, B and Russell, SJ (2008). Measles virus as an oncolytic vector platform. Curr Gene Ther 8: 162–175. 18537591 10.2174/156652308784746459

[bib109] Allen, C, Opyrchal, M, Aderca, I, Schroeder, MA, Sarkaria, JN, Domingo, E et al. (2013). Oncolytic measles virus strains have significant antitumor activity against glioma stem cells. Gene Ther 20: 444–449. 22914495 10.1038/gt.2012.62 PMC3509233

[bib110] Enders, JF, Katz, SL, Milovanovic, MV and Holloway, A (1960). Studies on an attenuated measles-virus vaccine. I. Development and preparations of the vaccine: technics for assay of effects of vaccination. N Engl J Med 263: 153–159. 13820246 10.1056/NEJM196007282630401

[bib111] Grote, D, Russell, SJ, Cornu, TI, Cattaneo, R, Vile, R, Poland, GA et al. (2001). Live attenuated measles virus induces regression of human lymphoma xenografts in immunodeficient mice. Blood 97: 3746–3754. 11389012 10.1182/blood.v97.12.3746

[bib112] Dingli, D, Peng, KW, Harvey, ME, Greipp, PR, O’Connor, MK, Cattaneo, R et al. (2004). Image-guided radiovirotherapy for multiple myeloma using a recombinant measles virus expressing the thyroidal sodium iodide symporter. Blood 103: 1641–1646. 14604966 10.1182/blood-2003-07-2233

[bib113] Studebaker, AW, Kreofsky, CR, Pierson, CR, Russell, SJ, Galanis, E and Raffel, C (2010). Treatment of medulloblastoma with a modified measles virus. Neuro Oncol 12: 1034–1042. 20494960 10.1093/neuonc/noq057 PMC3018921

[bib114] Phuong, LK, Allen, C, Peng, KW, Giannini, C, Greiner, S, TenEyck, CJ et al. (2003). Use of a vaccine strain of measles virus genetically engineered to produce carcinoembryonic antigen as a novel therapeutic agent against glioblastoma multiforme. Cancer Res 63: 2462–2469. 12750267

[bib115] Blechacz, B, Splinter, PL, Greiner, S, Myers, R, Peng, KW, Federspiel, MJ et al. (2006). Engineered measles virus as a novel oncolytic viral therapy system for hepatocellular carcinoma. Hepatology 44: 1465–1477. 17133484 10.1002/hep.21437

[bib116] Msaouel, P, Iankov, ID, Allen, C, Aderca, I, Federspiel, MJ, Tindall, DJ et al. (2009). Noninvasive imaging and radiovirotherapy of prostate cancer using an oncolytic measles virus expressing the sodium iodide symporter. Mol Ther 17: 2041–2048. 19773744 10.1038/mt.2009.218 PMC2810133

[bib117] McDonald, CJ, Erlichman, C, Ingle, JN, Rosales, GA, Allen, C, Greiner, SM et al. (2006). A measles virus vaccine strain derivative as a novel oncolytic agent against breast cancer. Breast Cancer Res Treat 99: 177–184. 16642271 10.1007/s10549-006-9200-5

[bib118] Iankov, ID, Msaouel, P, Allen, C, Federspiel, MJ, Bulur, PA, Dietz, AB et al. (2010). Demonstration of anti-tumor activity of oncolytic measles virus strains in a malignant pleural effusion breast cancer model. Breast Cancer Res Treat 122: 745–754. 19894113 10.1007/s10549-009-0602-z PMC2935656

[bib119] Peng, KW, Hadac, EM, Anderson, BD, Myers, R, Harvey, M, Greiner, SM et al. (2006). Pharmacokinetics of oncolytic measles virotherapy: eventual equilibrium between virus and tumor in an ovarian cancer xenograft model. Cancer Gene Ther 13: 732–738. 16543921 10.1038/sj.cgt.7700948

[bib120] Friedrich, K, Hanauer, JR, Prüfer, S, Münch, RC, Völker, I, Filippis, C et al. (2013). DARPin-targeting of measles virus: unique bispecificity, effective oncolysis, and enhanced safety. Mol Ther 21: 849–859. 23380817 10.1038/mt.2013.16 PMC3616535

[bib121] Msaouel, P, Iankov, ID, Allen, C, Russell, SJ and Galanis, E (2012). Oncolytic measles virus retargeting by ligand display. Methods Mol Biol 797: 141–162. 21948475 10.1007/978-1-61779-340-0_11 PMC3691680

[bib122] Msaouel, P, Dispenzieri, A and Galanis, E (2009). Clinical testing of engineered oncolytic measles virus strains in the treatment of cancer: an overview. Curr Opin Mol Ther 11: 43–53. 19169959 PMC2717625

[bib123] Msaouel, P, Iankov, ID, Dispenzieri, A and Galanis, E (2012). Attenuated oncolytic measles virus strains as cancer therapeutics. Curr Pharm Biotechnol 13: 1732–1741. 21740361 10.2174/138920112800958896 PMC3298624

[bib124] Hartkopf, AD, Bossow, S, Lampe, J, Zimmermann, M, Taran, FA, Wallwiener, D et al. (2013). Enhanced killing of ovarian carcinoma using oncolytic measles vaccine virus armed with a yeast cytosine deaminase and uracil phosphoribosyltransferase. Gynecol Oncol 130: 362–368. 23676551 10.1016/j.ygyno.2013.05.004

[bib125] Yurttas, C, Berchtold, S, Malek, NP, Bitzer, M and Lauer, UM (2014). Pulsed versus continuous application of the prodrug 5-fluorocytosine to enhance the oncolytic effectiveness of a measles vaccine virus armed with a suicide gene. Hum Gene Ther Clin Dev 25: 85–96. 24933569 10.1089/humc.2013.127

[bib126] Noll, M, Berchtold, S, Lampe, J, Malek, NP, Bitzer, M and Lauer, UM (2013). Primary resistance phenomena to oncolytic measles vaccine viruses. Int J Oncol 43: 103–112. 23612727 10.3892/ijo.2013.1914

[bib127] Lange, S, Lampe, J, Bossow, S, Zimmermann, M, Neubert, W, Bitzer, M et al. (2013). A novel armed oncolytic measles vaccine virus for the treatment of cholangiocarcinoma. Hum Gene Ther 24: 554–564. 23550539 10.1089/hum.2012.136 PMC3655633

[bib128] Völker, I, Bach, P, Coulibaly, C, Plesker, R, Abel, T, Seifried, J et al. (2013). Intrahepatic application of suicide gene-armed measles virotherapeutics: a safety study in transgenic mice and rhesus macaques. Hum Gene Ther Clin Dev 24: 11–22. 23692379 10.1089/humc.2012.242

[bib129] Tanemura, A, Kiyohara, E, Katayama, I and Kaneda, Y (2013). Recent advances and developments in the antitumor effect of the HVJ envelope vector on malignant melanoma: from the bench to clinical application. Cancer Gene Ther 20: 599–605. 24157924 10.1038/cgt.2013.61

[bib130] Slobod, KS, Shenep, JL, Luján-Zilbermann, J, Allison, K, Brown, B, Scroggs, RA et al. (2004). Safety and immunogenicity of intranasal murine parainfluenza virus type 1 (Sendai virus) in healthy human adults. Vaccine 22: 3182–3186. 15297072 10.1016/j.vaccine.2004.01.053

[bib131] Emmerson, PT (1999). Newcastle Disease Virus (Paramyxoviridae) Encyclopedia of Virology. Elsevier: Oxford, pp. 1020–1026.

[bib132] Swayne, DE and King, DJ (2003). Avian influenza and Newcastle disease. J Am Vet Med Assoc 222: 1534–1540. 12784958 10.2460/javma.2003.222.1534

[bib133] Csatary, LK, Eckhardt, S, Bukosza, I, Czegledi, F, Fenyvesi, C, Gergely, P et al. (1993). Attenuated veterinary virus vaccine for the treatment of cancer. Cancer Detect Prev 17: 619–627. 8275514

[bib134] Csatary, LK, Gosztonyi, G, Szeberenyi, J, Fabian, Z, Liszka, V, Bodey, B et al. (2004). MTH-68/H oncolytic viral treatment in human high-grade gliomas. J Neurooncol 67: 83–93. 15072452 10.1023/b:neon.0000021735.85511.05

[bib135] Freeman, AI, Zakay-Rones, Z, Gomori, JM, Linetsky, E, Rasooly, L, Greenbaum, E et al. (2006). Phase I/II trial of intravenous NDV-HUJ oncolytic virus in recurrent glioblastoma multiforme. Mol Ther 13: 221–228. 16257582 10.1016/j.ymthe.2005.08.016

[bib136] Lorence, RM, Roberts, MS, O’Neil, JD, Groene, WS, Miller, JA, Mueller, SN et al. (2007). Phase 1 clinical experience using intravenous administration of PV701, an oncolytic Newcastle disease virus. Curr Cancer Drug Targets 7: 157–167. 17346107 10.2174/156800907780058853

[bib137] Hotte, SJ, Lorence, RM, Hirte, HW, Polawski, SR, Bamat, MK, O’Neil, JD et al. (2007). An optimized clinical regimen for the oncolytic virus PV701. Clin Cancer Res 13: 977–985. 17289893 10.1158/1078-0432.CCR-06-1817

[bib138] Cassel, WA and Murray, DR (1992). A ten-year follow-up on stage II malignant melanoma patients treated postsurgically with Newcastle disease virus oncolysate. Med Oncol Tumor Pharmacother 9: 169–171. 1342060 10.1007/BF02987752

[bib139] Batliwalla, FM, Bateman, BA, Serrano, D, Murray, D, Macphail, S, Maino, VC et al. (1998). A 15-year follow-up of AJCC stage III malignant melanoma patients treated postsurgically with Newcastle disease virus (NDV) oncolysate and determination of alterations in the CD8 T cell repertoire. Mol Med 4: 783–794. 9990864 PMC2230393

[bib140] Karcher, J, Dyckhoff, G, Beckhove, P, Reisser, C, Brysch, M, Ziouta, Y et al. (2004). Antitumor vaccination in patients with head and neck squamous cell carcinomas with autologous virus-modified tumor cells. Cancer Res 64: 8057–8061. 15520216 10.1158/0008-5472.CAN-04-1545

[bib141] Herold-Mende, C, Karcher, J, Dyckhoff, G and Schirrmacher, V (2005). Antitumor immunization of head and neck squamous cell carcinoma patients with a virus-modified autologous tumor cell vaccine. Adv Otorhinolaryngol 62: 173–183. 15608427 10.1159/000082507

[bib142] Schulze, T, Kemmner, W, Weitz, J, Wernecke, KD, Schirrmacher, V and Schlag, PM (2009). Efficiency of adjuvant active specific immunization with Newcastle disease virus modified tumor cells in colorectal cancer patients following resection of liver metastases: results of a prospective randomized trial. Cancer Immunol Immunother 58: 61–69. 18488223 10.1007/s00262-008-0526-1 PMC11030620

[bib143] Schirrmacher, V and Fournier, P (2009). Newcastle disease virus: a promising vector for viral therapy, immune therapy, and gene therapy of cancer. Methods Mol Biol 542: 565–605. 19565923 10.1007/978-1-59745-561-9_30 PMC7122391

[bib144] Pecora, AL, Rizvi, N, Cohen, GI, Meropol, NJ, Sterman, D, Marshall, JL et al. (2002). Phase I trial of intravenous administration of PV701, an oncolytic virus, in patients with advanced solid cancers. J Clin Oncol 20: 2251–2266. 11980996 10.1200/JCO.2002.08.042

[bib145] Schirrmacher, V, Griesbach, A and Ahlert, T (2001). Antitumor effects of Newcastle disease virus *in vivo*: local versus systemic effects. Int J Oncol 18: 945–952. 11295039 10.3892/ijo.18.5.945

[bib146] Lorence, RM, Pecora, AL, Major, PP, Hotte, SJ, Laurie, SA, Roberts, MS et al. (2003). Overview of phase I studies of intravenous administration of PV701, an oncolytic virus. Curr Opin Mol Ther 5: 618–624. 14755888

[bib147] Laurie, SA, Bell, JC, Atkins, HL, Roach, J, Bamat, MK, O’Neil, JD et al. (2006). A phase 1 clinical study of intravenous administration of PV701, an oncolytic virus, using two-step desensitization. Clin Cancer Res 12: 2555–2562. 16638865 10.1158/1078-0432.CCR-05-2038

[bib148] Guillerme, J-B, Gregoire, M, Tangy, F and Fonteneau J-F (2013). Antitumor virotherapy by attenuated measles virus. Biology 2: 587–602. 24832799 10.3390/biology2020587 PMC3960896

[bib149] Heinzerling, L, Künzi, V, Oberholzer, PA, Kündig, T, Naim, H and Dummer, R (2005). Oncolytic measles virus in cutaneous T-cell lymphomas mounts antitumor immune responses *in vivo* and targets interferon-resistant tumor cells. Blood 106: 2287–2294. 15961518 10.1182/blood-2004-11-4558

[bib150] Galanis, E, Hartmann, LC, Cliby, WA, Long, HJ, Peethambaram, PP, Barrette, BA et al. (2010). Phase I trial of intraperitoneal administration of an oncolytic measles virus strain engineered to express carcinoembryonic antigen for recurrent ovarian cancer. Cancer Res 70: 875–882. 20103634 10.1158/0008-5472.CAN-09-2762 PMC2890216

[bib151] Russell, SJ, Federspiel, MJ, Peng, KW, Tong, C, Dingli, D, Morice, WG et al. (2014). Remission of disseminated cancer after systemic oncolytic virotherapy. Mayo Clin Proc 89: 926–933. 24835528 10.1016/j.mayocp.2014.04.003 PMC4225126

[bib152] Galanis, E, Atherton, PJ, Maurer, MJ, Knutson, KL, Dowdy, SC, Cliby, WA et al. (2015). Oncolytic measles virus expressing the sodium iodide symporter to treat drug-resistant ovarian cancer. Cancer Res 75: 22–30. 25398436 10.1158/0008-5472.CAN-14-2533 PMC4377302

[bib153] Shimizu, Y, Hasumi, K, Okudaira, Y, Yamanishi, K and Takahashi, M (1988). Immunotherapy of advanced gynecologic cancer patients utilizing mumps virus. Cancer Detect Prev 12: 487–495. 2972361

[bib154] Asada, T (1974). Treatment of human cancer with mumps virus. Cancer 34: 1907–1928. 4611607 10.1002/1097-0142(197412)34:6<1907::aid-cncr2820340609>3.0.co;2-4

[bib155] Okuno, Y, Asada, T, Yamanishi, K, Otsuka, T, Takahashi, M, Tanioka, T et al. (1978). Studies on the use of mumps virus for treatment of human cancer. Biken J 21: 37–49. 749908

[bib156] Grossardt, C, Engeland, CE, Bossow, S, Halama, N, Zaoui, K, Leber, MF et al. (2013). Granulocyte-macrophage colony-stimulating factor-armed oncolytic measles virus is an effective therapeutic cancer vaccine. Hum Gene Ther 24: 644–654. 23642239 10.1089/hum.2012.205 PMC3719441

[bib157] Weber, J (2007). Review: anti-CTLA-4 antibody ipilimumab: case studies of clinical response and immune-related adverse events. Oncologist 12: 864–872. 17673617 10.1634/theoncologist.12-7-864

[bib158] Barbee, MS, Ogunniyi, A, Horvat, TZ and Dang, TO (2015). Current status and future directions of the immune checkpoint inhibitors ipilimumab, pembrolizumab, and nivolumab in oncology. Ann Pharmacother 49: 907–937. 25991832 10.1177/1060028015586218

[bib159] Zamarin, D, Holmgaard, RB, Subudhi, SK, Park, JS, Mansour, M, Palese, P et al. (2014). Localized oncolytic virotherapy overcomes systemic tumor resistance to immune checkpoint blockade immunotherapy. Sci Transl Med 6: 226ra32. 10.1126/scitranslmed.3008095 PMC4106918 24598590

[bib160] Engeland, CE, Grossardt, C, Veinalde, R, Bossow, S, Lutz, D, Kaufmann, JK et al. (2014). CTLA-4 and PD-L1 checkpoint blockade enhances oncolytic measles virus therapy. Mol Ther 22: 1949–1959. 25156126 10.1038/mt.2014.160 PMC4429737

[bib161] Baertsch, MA, Leber, MF, Bossow, S, Singh, M, Engeland, CE, Albert, J et al. (2014). MicroRNA-mediated multi-tissue detargeting of oncolytic measles virus. Cancer Gene Ther 21: 373–380. 25145311 10.1038/cgt.2014.40

[bib162] Springfeld, C, von Messling, V, Frenzke, M, Ungerechts, G, Buchholz, CJ and Cattaneo, R (2006). Oncolytic efficacy and enhanced safety of measles virus activated by tumor-secreted matrix metalloproteinases. Cancer Res 66: 7694–7700. 16885371 10.1158/0008-5472.CAN-06-0538

[bib163] Coppola, JM, Bhojani, MS, Ross, BD and Rehemtulla, A (2008). A small-molecule furin inhibitor inhibits cancer cell motility and invasiveness. Neoplasia 10: 363–370. 18392131 10.1593/neo.08166 PMC2288536

[bib164] Bassi, DE, Mahloogi, H and Klein-Szanto, AJ (2000). The proprotein convertases furin and PACE4 play a significant role in tumor progression. Mol Carcinog 28: 63–69. 10900462

[bib165] Bassi, DE, Lopez De Cicco, R, Mahloogi, H, Zucker, S, Thomas, G and Klein-Szanto, AJ (2001). Furin inhibition results in absent or decreased invasiveness and tumorigenicity of human cancer cells. Proc Natl Acad Sci USA 98: 10326–10331. 11517338 10.1073/pnas.191199198 PMC56960

[bib166] Bassi, DE, Mahloogi, H, Al-Saleem, L, Lopez De Cicco, R, Ridge, JA and Klein-Szanto, AJ (2001). Elevated furin expression in aggressive human head and neck tumors and tumor cell lines. Mol Carcinog 31: 224–232. 11536372 10.1002/mc.1057

[bib167] Bassi, DE, Mahloogi, H, Lopez De Cicco, R and Klein-Szanto, A (2003). Increased furin activity enhances the malignant phenotype of human head and neck cancer cells. Am J Pathol 162: 439–447. 12547702 10.1016/s0002-9440(10)63838-2 PMC1851171

[bib168] Fu, J, Bassi, DE, Zhang, J, Li, T, Nicolas, E and Klein-Szanto, AJ (2012). Transgenic overexpression of the proprotein convertase furin enhances skin tumor growth. Neoplasia 14: 271–282. 22577343 10.1593/neo.12166 PMC3349254

[bib169] Zamarin, D, Vigil, A, Kelly, K, García-Sastre, A and Fong, Y (2009). Genetically engineered Newcastle disease virus for malignant melanoma therapy. Gene Ther 16: 796–804. 19242529 10.1038/gt.2009.14 PMC2882235

[bib170] Silberhumer, GR, Brader, P, Wong, J, Serganova, IS, Gönen, M, Gonzalez, SJ et al. (2010). Genetically engineered oncolytic Newcastle disease virus effectively induces sustained remission of malignant pleural mesothelioma. Mol Cancer Ther 9: 2761–2769. 20858727 10.1158/1535-7163.MCT-10-0090 PMC3266818

[bib171] Li, P, Chen, CH, Li, S, Givi, B, Yu, Z, Zamarin, D et al. (2011). Therapeutic effects of a fusogenic newcastle disease virus in treating head and neck cancer. Head Neck 33: 1394–1399. 21928411 10.1002/hed.21609 PMC3116983

[bib172] Peeters, BP, de Leeuw, OS, Koch, G and Gielkens, AL (1999). Rescue of Newcastle disease virus from cloned cDNA: evidence that cleavability of the fusion protein is a major determinant for virulence. J Virol 73: 5001–5009. 10233962 10.1128/jvi.73.6.5001-5009.1999 PMC112544

[bib173] Elankumaran, S, Rockemann, D and Samal, SK (2006). Newcastle disease virus exerts oncolysis by both intrinsic and extrinsic caspase-dependent pathways of cell death. J Virol 80: 7522–7534. 16840332 10.1128/JVI.00241-06 PMC1563725

[bib174] Altomonte, J and Ebert, O (2012). Replicating viral vectors for cancer therapy: strategies to synergize with host immune responses. Microb Biotechnol 5: 251–259. 21923638 10.1111/j.1751-7915.2011.00296.x PMC3815785

[bib175] Kinoh, H, Inoue, M, Washizawa, K, Yamamoto, T, Fujikawa, S, Tokusumi, Y et al. (2004). Generation of a recombinant Sendai virus that is selectively activated and lyses human tumor cells expressing matrix metalloproteinases. Gene Ther 11: 1137–1145. 15085175 10.1038/sj.gt.3302272

[bib176] Kinoh, H, Inoue, M, Komaru, A, Ueda, Y, Hasegawa, M and Yonemitsu, Y (2009). Generation of optimized and urokinase-targeted oncolytic Sendai virus vectors applicable for various human malignancies. Gene Ther 16: 392–403. 19037241 10.1038/gt.2008.167

[bib177] Zimmermann, M, Armeanu-Ebinger, S, Bossow, S, Lampe, J, Smirnow, I, Schenk, A et al. (2014). Attenuated and protease-profile modified sendai virus vectors as a new tool for virotherapy of solid tumors. PLoS One 9: e90508. 24598703 10.1371/journal.pone.0090508 PMC3944018

[bib178] Thompson, JD, Higgins, DG and Gibson, TJ (1994). CLUSTAL W: improving the sensitivity of progressive multiple sequence alignment through sequence weighting, position-specific gap penalties and weight matrix choice. Nucleic Acids Res 22: 4673–4680. 7984417 10.1093/nar/22.22.4673 PMC308517

[bib179] Tamura, K, Peterson, D, Peterson, N, Stecher, G, Nei, M and Kumar, S (2011). MEGA5: molecular evolutionary genetics analysis using maximum likelihood, evolutionary distance, and maximum parsimony methods. Mol Biol Evol 28: 2731–2739. 21546353 10.1093/molbev/msr121 PMC3203626

[bib180] de Leeuw, O and Peeters, B (1999). Complete nucleotide sequence of Newcastle disease virus: evidence for the existence of a new genus within the subfamily Paramyxovirinae. J Gen Virol 80: 131–136. 9934695 10.1099/0022-1317-80-1-131

[bib181] Steiner, HH, Bonsanto, MM, Beckhove, P, Brysch, M, Geletneky, K, Ahmadi, R et al. (2004). Antitumor vaccination of patients with glioblastoma multiforme: a pilot study to assess feasibility, safety, and clinical benefit. J Clin Oncol 22: 4272–4281. 15452186 10.1200/JCO.2004.09.038

